# Autoproteolysis and Intramolecular Dissociation of *Yersinia* YscU Precedes Secretion of Its C-Terminal Polypeptide YscU_CC_

**DOI:** 10.1371/journal.pone.0049349

**Published:** 2012-11-21

**Authors:** Stefan Frost, Oanh Ho, Frédéric H. Login, Christoph F. Weise, Hans Wolf-Watz, Magnus Wolf-Watz

**Affiliations:** 1 Department of Molecular Biology and The Laboratory for Molecular Infection Medicine Sweden (MIMS), Umeå University, Umeå, Sweden; 2 Department of Chemistry, Chemical Biological Center, Umeå University, Umeå, Sweden; Centre National de la Recherche Scientifique, Aix-Marseille Université, France

## Abstract

Type III secretion system mediated secretion and translocation of Yop-effector proteins across the eukaryotic target cell membrane by pathogenic *Yersinia* is highly organized and is dependent on a switching event from secretion of early structural substrates to late effector substrates (Yops). Substrate switching can be mimicked *in vitro* by modulating the calcium levels in the growth medium. YscU that is essential for regulation of this switch undergoes autoproteolysis at a conserved N↑PTH motif, resulting in a 10 kDa C-terminal polypeptide fragment denoted YscU_CC_. Here we show that depletion of calcium induces intramolecular dissociation of YscU_CC_ from YscU followed by secretion of the YscU_CC_ polypeptide. Thus, YscU_CC_ behaved *in vivo* as a Yop protein with respect to secretion properties. Further, destabilized *ysc*U mutants displayed increased rates of dissociation of YscU_CC_ *in vitro* resulting in enhanced Yop secretion *in vivo* at 30°C relative to the wild-type strain.These findings provide strong support to the relevance of YscU_CC_ dissociation for Yop secretion. We propose that YscU_CC_ orchestrates a block in the secretion channel that is eliminated by calcium depletion. Further, the striking homology between different members of the YscU/FlhB family suggests that this protein family possess regulatory functions also in other bacteria using comparable mechanisms.

## Introduction

In 1952, Hills and Spurr showed that virulent strains of *Yersinia pestis* (*Pasturella pestis*) were unable to grow and divide when incubated at 37°C; instead, they required incubation at 27°C [1]. This phenotype was surprising, because *Y. pestis* causes lethal infections in rodents and humans, which have a body temperature close to 37°C. Moreover, no typical nutritional requirements could explain this phenotype. Later, Kupferberg and Smith demonstrated that addition of 2.5 mM calcium to the growth medium supported growth of *Y. pestis* at 37°C [2]. This unusual requirement for calcium was later shown to be correlated to the massive synthesis and secretion of a number of proteins, called *Yersinia* outer proteins (Yops). This was based on the observation that 2.5 mM calcium in the growth medium blocked Yop secretion, while depletion of calcium induced massive Yop secretion that also results in stop of bacteria proliferation [3], [4], [5]. Synthesis and secretion of Yops are dependent on a virulence plasmid [6], a common feature of all human pathogenic *Yersinia* (*Y. pestis*, *Y. enterocolitica*, and *Y. pseudotuberculosis*). Yops are synthesized during infection, which indicates their importance in virulence [3]. Yop secretion involves the type III secretion system (T3SS) of *Yersinia*, which is encoded by the same virulence plasmid that carries the *yop* genes. The T3SS is a dedicated secretion system that forms a multi-protein complex of around 25 proteins spanning the inner and outer bacterial membranes [7]. It is built up by a basal body located in the membrane showing high homology with a corresponding structure of the bacterial flagellum. A needle is anchored to the basal body forming hollow tube measuring around 60 to 80 nm in length and 8 nm in external width with an inner diameter of 3 nm [8], [9]. It has been postulated that Yops are transferred to the target cell through the needle structure [8]. This model has however been challenged in recent work from our laboratory [10] where we show that bacterial surface localized Yop-effectors can be translocated into the target cell. Hence, translocation can occur via a mechanism that is distinct from the postulated micro-injection model. *Yersinia* employs the T3SS to secrete Yops into the external environment and to translocate Yops into the cytoplasm of eukaryotic target cells [11]. These processes are highly regulated. It has been shown that *Y. pseudotuberculosis* up-regulates *yop* expression after contact with eukaryotic cells, and this requires a functional T3SS [12], [13]. Importantly, target cell contact can be mimicked by depleting calcium in the growth medium and simultaneously shifting the temperature from 26°C to 37°C [11]. Modulation of calcium levels in the growth medium has been an invaluable tool for increasing our understanding of T3SSs in *Yersinia* virulence. Several seminal and general discoveries have been made based on the calcium effect, including T3SS mediated secretion, translocation, and target cell induced expression of effector proteins [12], [13], [14].

**Figure 1:**
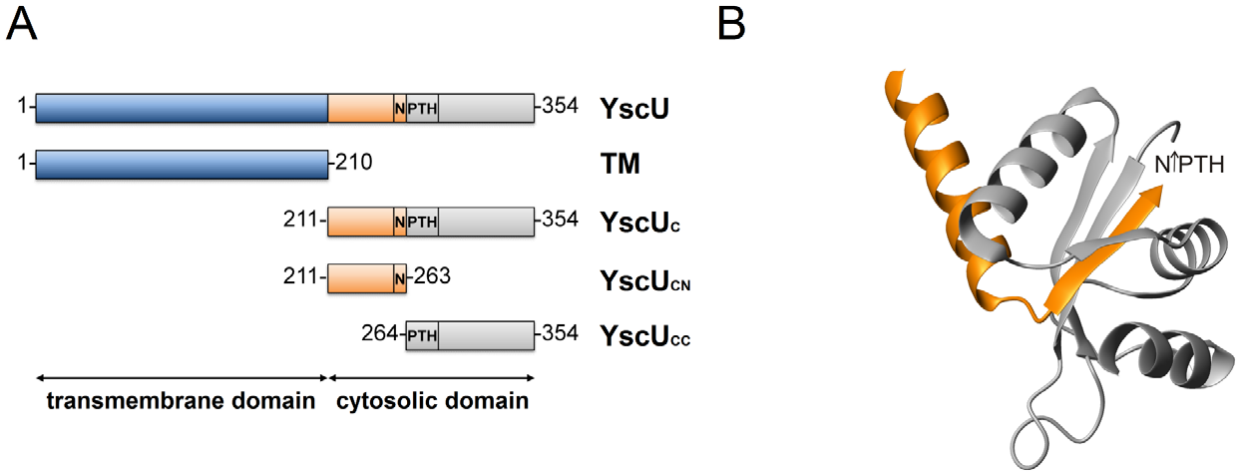
Domain structure of YscU and crystallographic structure of YscU_C_. (**A**) Schematic domain structure of the integral membrane protein, YscU of *Y. pseudotuberculosis*. The full-length protein contains 354 amino acid residues. The N-terminal 210 residues constitute four transmembrane helices (TM). The cytosolic domain of YscU (YscU_C_) undergoes autoproteolytic cleavage at the N↑PTH-motif (amino acids 263–266), which leaves an N-terminal cytoplasmic polypeptide, denoted YscU_CN_, and a C-terminal polypeptide, denoted YscU_CC_. (**B**) Ribbon drawing of the cleaved cytosolic domain YscU_C_ (2JLI.PDB) from *Y. pestis* [39]. YscU_CN_ and YscU_CC_ resulting from cleavage at the N↑PTH motif are colored in orange and grey, respectively.

The YscU protein of *Yersinia* is an integral inner-membrane protein with four membrane spanning segments (Figure 1A) and is required for T3SS function. It belongs to a family of proteins (YscU/FlhB class) that is characterized by auto-cleavage at a highly conserved N↑PTH motif (amino acids 263–266) [15]. Autoproteolysis of YscU is required for proper regulation of Yops synthesis and secretion. Furthermore, the Yop synthesis and secretion is lost when the full *ysc*U gene or the N↑PTH coding sequence are deleted, indicating the importance of YscU for T3SS function. Similar phenotypes are observed when point mutations affect cleavage at the N↑PTH motif; this illustrates the importance of cleavage for calcium regulation [16], [17], [18], [19]. Full length YscU (denoted YscU) contains two domains, the transmembrane domain (TM) and a soluble cytoplasmic domain, denoted YscU_C_ (Figure 1A and Figure 1B). Autoproteolysis of YscU occurs between asparagine 263 and proline 264 at the N↑PTH motif and results in a 10 kDa C-terminal polypeptide fragment, denoted YscU_CC_ that is attached to the remainder of the protein through protein-protein interactions. In context of the cytoplasmic domain, YscU_C_ (which is used extensively in this article), cleavage generates two fragments; the YscU_CC_ fragment and a 6 kDa N-terminal fragment denoted YscU_CN_ [16], [18] (Figure 1A).

Both YscP (FliK) and YscU (FlhB) have been linked to the “substrate specificity switch”, first identified by MacNab and coworkers in the flagellum T3SS [15], [20]. This switching machinery changes secretion specificity from early hook substrates to late filament substrates as one step in the assembly of the flagellum [21]. It has been suggested that the C-terminal domain of FliK (FliK_C_) binds to the C-terminal cytosolic domain of FlhB (FlhB_C_), causing a conformational change in FlhB_C_ that is required for the switch [20]. An *ysc*P mutant was impaired in switching from the early secretion of needle subunits (YscF) to the late export of Yops [16], [22]. This led to a phenotype with unusually long needles unable to secrete Yop proteins, thus YscP is an essential protein for T3SS mediated secretion [16], [22], [23]. A similar phenotype was observed for the *ysc*U mutant, *N263A*, which highlighted the importance of YscU autoproteolysis in the substrate specificity switch [16]. Interestingly, an *ysc*P null mutant was suppressed by single amino acid substitutions in YscU_C_, and these suppressor mutants partially restored Yop secretion [17]. This suggested that YscU and YscP interact, and that this interaction was essential for proper control of needle formation and Yop secretion [16]. A direct interaction between the YscU and YscP orthologs, Flik and FlhB has been shown with surface plasmon resonance experiments [24]. In analogy, mutations in the corresponding *ysc*P gene in *Shigella flexneri* (*spa32*) and *Salmonella thypimurium* (*inv*J) [25] also caused defective substrate switching. It has been shown that Spa32 (YscP) and Spa40 (YscU) interact [26], [27]. Given the high functional similarity between the T3SSs of different species, it is likely that the substrate specificity switch is regulated by a similar mechanism in different pathogens.

Here, we studied the functional role of YscU in Yop secretion by exploiting the calcium regulation of substrate switching in *Y. pseudotuberculosis*. We combined *in vivo* and *in vitro* methods to examine the steps of YscU autoproteolysis, subsequent dissociation, and secretion of YscU_CC_ and how they affect Yop secretion in *Y. pseudotuberculosis* during growth in calcium depleted media.

**Figure 2:**
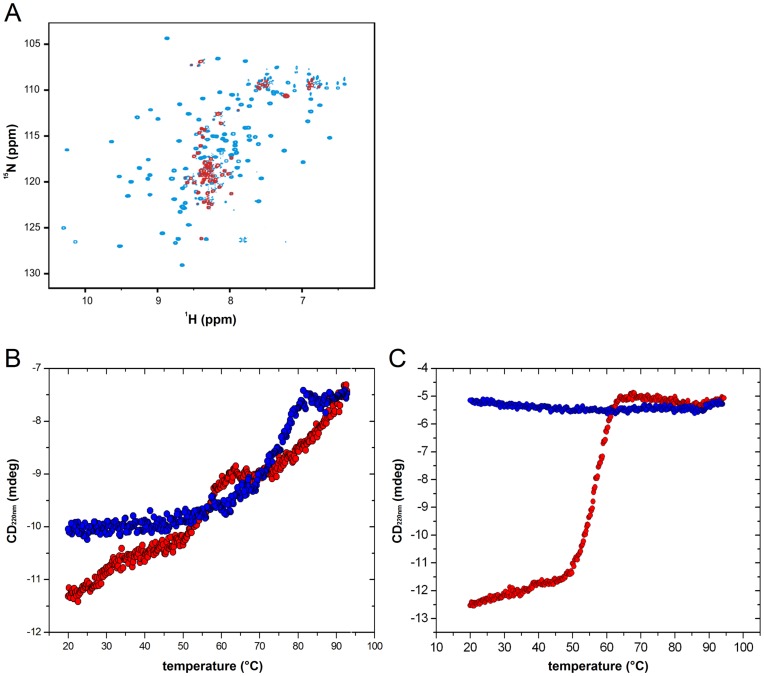
*In vitro* dissociation of YscU_C_. (**A**) ^1^H-^15^N HSQC spectra of YscU_C_ at 20°C, pH 7.4, before (blue contours) and after (red contours) incubation at 60°C for 10 min. Only resonances that corresponded to YscU_CN_ were visible after the thermal treatment. (**B**) Thermal up- and down-scans of YscU_C_ at pH 7.4 monitored with CD spectroscopy at 220 nm in the absence of calcium. (**C**) Thermal signatures of P264A, a non-cleavable mutant, at pH 7.4 in the absence of calcium. Up- and down scans of YscU_C_ and P264A are shown in red and blue circles, respectively.

## Materials and Methods

### Bacterial Strains, Plasmids, and Growth Conditions

Bacterial strains and plasmids used in this study are listed in the supporting material (“Table S1”). *Escherichia coli* strains were grown in Luria-Bertani broth (LB) or on Luria agar plates at 37°C. *Y. pseudotuberculosis* was grown at either 26°C or 37°C in Hepes buffered LB or on Luria agar plates (unless specified in the text). Antibiotics were used for selection according to the resistance markers carried by the strains at the following concentrations: kanamycin, 50 *µ*g/ml; chloramphenicol, 25 *µ*g/ml; and carbenicillin, 100 *µ*g/ml. EGTA was added to the media at a final concentration of 5 mM and 20 mM MgCl_2_ to create calcium depleted conditions.

**Figure 3:**
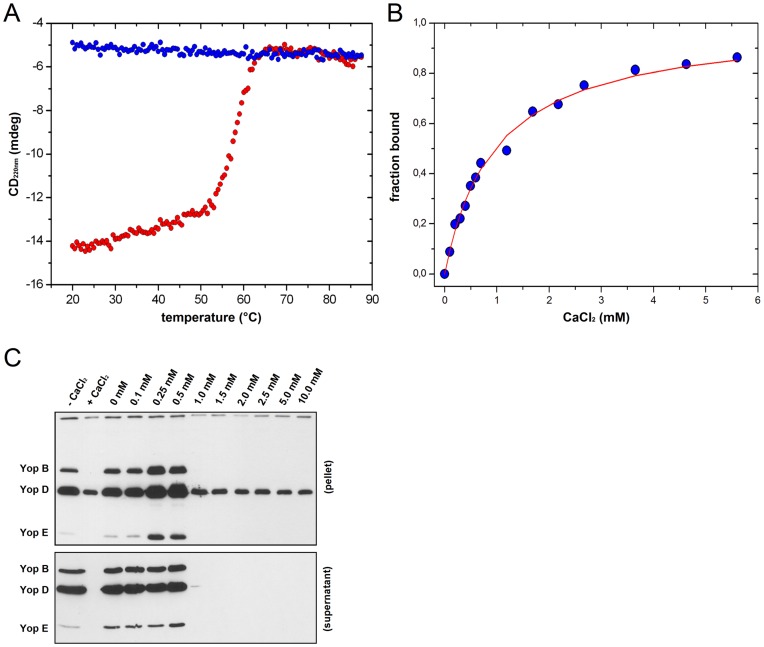
Calcium effects on YscU_C_ stability and Yop secretion. (**A**) Thermal up- and down-scans of YscU_C_ at pH 7.4 monitored with CD spectroscopy at 220 nm in the presence of 2.5 mM calcium. Up- and down scans of YscU_C_ are shown in red and blue circles, respectively. (**B**) Titration of calcium to YscU_C_ monitored with CD spectroscopy at 220 nm. The calcium binding isotherm to YscU_C_ was fit to a one-site binding model (red line). The resulting *K_d_* was 800 µM. (**C**) Western Blot analysis of YopB, YopD, and YopE in wild-type *Y. pseudotuberculosis*. The bacteria were grown for 2 h at 26°C and shifted to 37°C for 3 h (temperature shift for induction of Yop secretion) with varying concentrations of free calcium. “Pellet” indicates intracellular proteins; “supernatant” denotes secreted proteins. The LB growth medium was initially supplemented with 1 mM EGTA to complex residual calcium content (approximately 500 µM); thereafter, calcium was added to set the indicated concentrations of free calcium.

### Yop Secretion Assay

Cultures were started at an absorbance of OD_600_ = 0.1 in Hepes buffered LB with the appropriate antibiotics. Bacteria were grown at 26°C for 2 h and shifted to 37°C for 3 h in calcium-supplemented or calcium-depleted conditions (except where specified in the text). Cultures were harvested and centrifuged for 10 min at 4 000×*g*. Aliquots (4.5 ml) of filtrated supernatant were combined with 10% (v/v) trichloroacetic acid (TCA) for protein precipitation. Precipitated proteins were solubilized in SDS-PAGE loading buffer. The pelleted cells were resuspended in an equal volume of LB and lysed with SDS-PAGE loading buffer. Cells and supernatants were loaded at equivalent protein concentrations (according to OD_600_) and separated by SDS-PAGE. Proteins were either stained with Coomassie R250 or, alternatively, transferred onto a PVDF membrane (GE Healthcare) for immunoblotting. Anti-Yop antibodies were diluted at 1∶5 000 and horseradish peroxidase-conjugated anti-rabbit IgG was diluted at 1∶10 000 (GE Healthcare). Proteins were detected with a chemiluminescence detection kit (GE Healthcare).

**Table 1:** Dissociation temperatures of YscU_C_ in presence of different divalent cations and *in vitro* binding affinities.

	CaCl_2_		BaCl_2_	SrCl_2_		MgCl_2_
Dissociationtemperature, *T_diss_*						
*T_diss_* (°C)	58.5±0.8		57.1±0.3	55.8±0.1		57.9±0.1
Dissociation constant, *K_d_*						
*K_d_* (µM)	800±40		900±140	840±100		130±10

CD spectroscopy at 220 nm was used to monitor thermal up- and down-scans of YscU_C_ in presence of different divalent cations at a scan rate of 1°C/min to determine dissociation temperatures (*T*_diss_, compare Figure 3A). To measure the binding isotherms (*K_d_*) of different divalent cations towards YscU_C_ (compare Figure 3B) titrations monitored with CD spectroscopy at 220 nm were performed.

### YscU_CC_ Overexpression and Secretion Assay

We grew YPIII/pIB102 bacterial strains, which contained the pBADmycHis B plasmid (Invitrogen) with the *yscU_CC_* expression sequence (see supporting “Material and methods S1”), and control strains contained an empty vector. The growth conditions were as described above, except 0.2% (v/v) of L-arabinose was added after 1 h at 26°C to induce biosynthesis of YscU_CC_. After separation by SDS-PAGE, proteins were stained with Coomassie R250 (Yop secretion) or transferred onto a PVDF membrane (GE Healthcare) for immunoblotting. Anti-YscU_CC_ peptide antibodies were diluted at 1∶5 000 [16] and horseradish peroxidase-conjugated anti-rabbit IgG was diluted at 1∶10 000 (GE Healthcare). Proteins were detected with a chemiluminescence detection kit (GE Healthcare).

**Figure 4:**
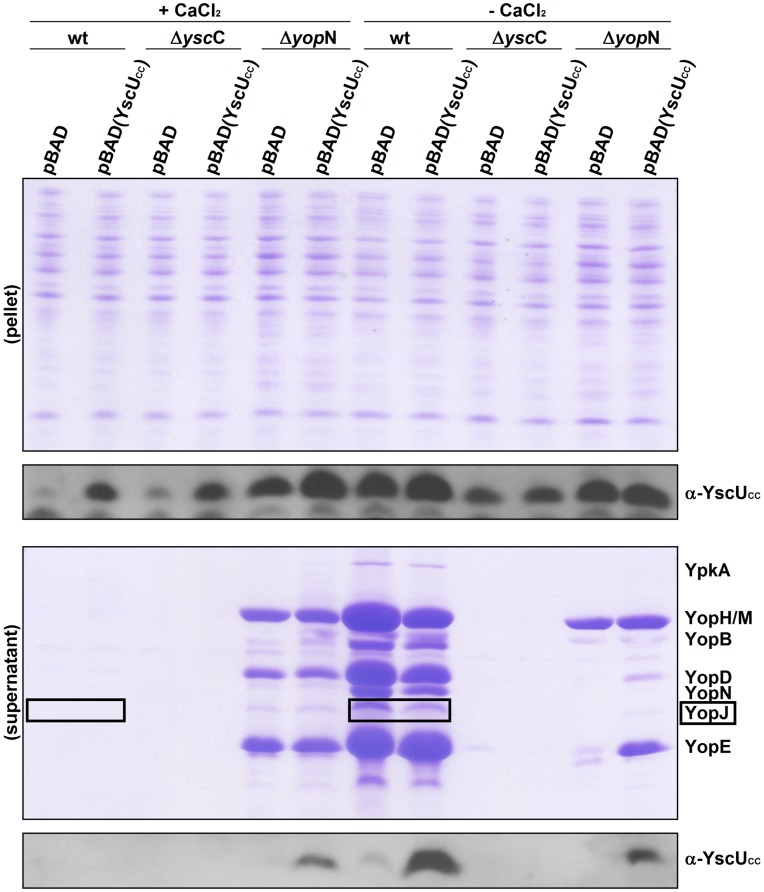
*In vivo* dissociation and secretion of YscU_CC_ in different *Yersinia* strains. Calcium dependent regulation of Yop and YscU_CC_ secretion in wild-type *Y. pseudotuberculosis*, in a Δ*ysc*C mutant and Δ*ysc*N mutant strain without and with *in trans* complementation of YscU_CC_. Bacteria transformed with empty pBADmycHis B (pBAD), or pBAD with one additional *yscU_CC_* copy (pBAD(YscU_CC_)), were grown for 2 h at 26°C and 3 h at 37°C in calcium depleted (−) or calcium supplemented (+) medium. The expression of *yscU_CC_* was induced by addition of arabinose. Yop secretion is coupled to the secretion of YscU_CC_ in all analysed *Yersinia* strains and required a functional T3SS. Secreted Yops visualized on Coomassie stained PAGE gels; YscU_CC_ visualized on immunoblots with anti-YscU_CC_ peptide antibodies. “pellet” indicates intracellular proteins; “supernatant” denotes secreted proteins. The YopJ protein (black box) was subjected to densitometric analysis for quantification of secretion levels (see Table 2).

**Table 2:** Comparative densitometric analysis of pH and calcium-dependent Yops secretion in *Y. pseudotuberculosis*.

condition	observed growth attenuation	observed Yop secretion	secretion efficiency (%)
pBAD, +Ca^2+^	no	no	0
pBAD/YscU_CC_), +Ca^2+^	no	no	0
pBAD, −Ca^2+^	yes (+++)	yes (+++)	99
pBAD/YscU_CC_), −Ca^2+*a*^	yes (+++)	yes (+++)	100
pH 6.0	yes (+)	yes (+)	1
pH 6.5	yes (++)	yes (++)	32
pH 7.0	yes (+++)	yes (+++)	75
pH 7.5^b^	yes (+++)	yes (+++)	100

**Figure 5:**
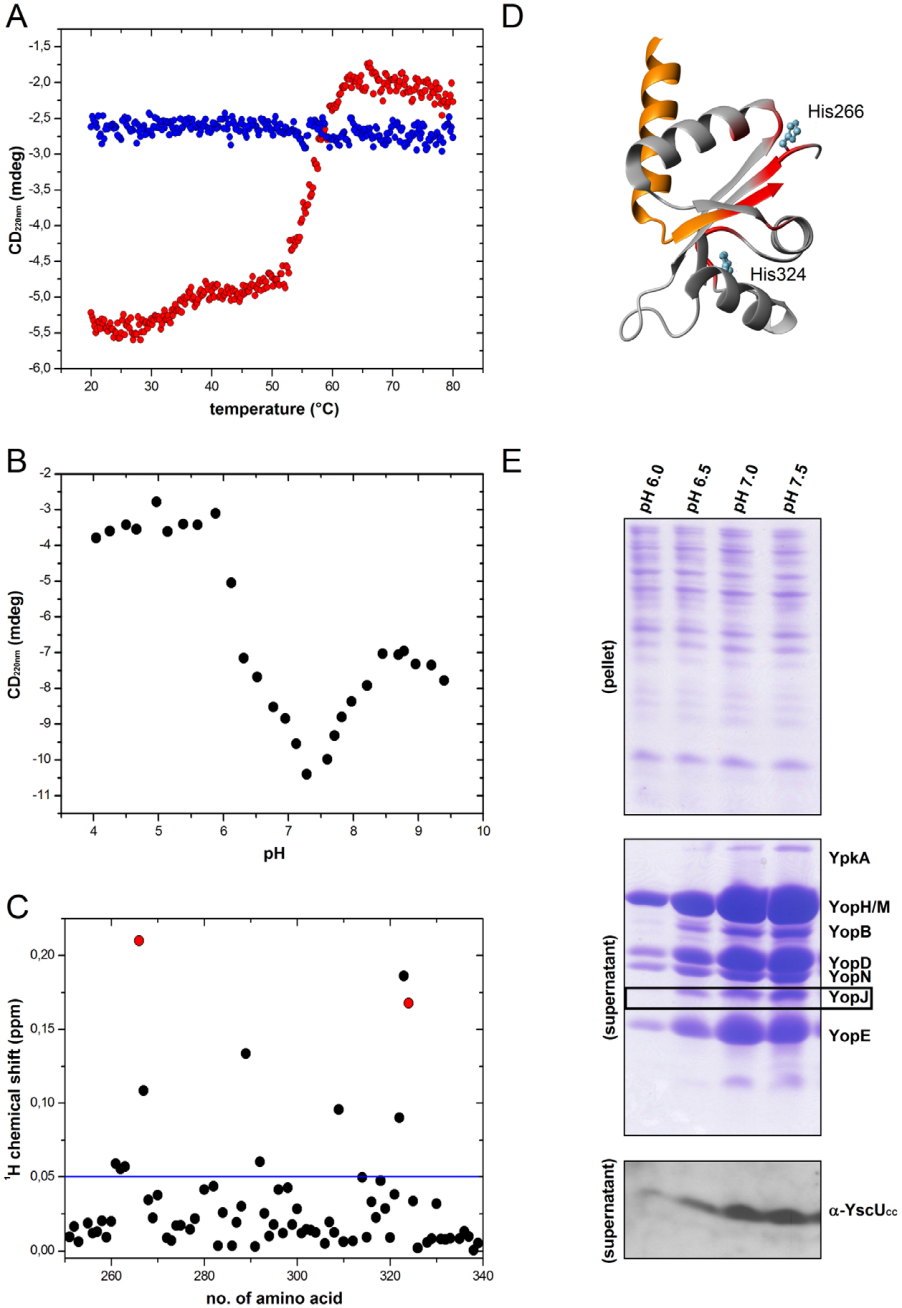
pH-dependencies of YscU_C_ dissociation *in vitro* and Yop/YscU_CC_ secretion *in vivo*. (**A**) Thermal up- and down-scans of YscU_C_ at pH 6.0, monitored with CD spectroscopy at 220 nm in the absence of calcium. The thermal signature of YscU_C_ displayed one large-amplitude transition at 55°C. Up- and down scans of YscU_C_ are shown in red and blue circles, respectively. (**B**) The pH-dependency of the YscU_C_ monitored with CD spectroscopy at 220 nm. (**C**) Chemical shift perturbations of YscU_C_ quantified from ^1^H-^15^N HSQC spectra, in response to a pH-shift from 7.4 to 6.0, displayed against the primary sequence. The blue line indicates the threshold value (0.05 ppm) used in Figure 5D. The chemical shift perturbation of the two histidines at positions 266 and 324 are shown in red. (**D**) Structural distributions of residues that show significant chemical shift perturbations in response to a pH-shift from 7.4 to 6.0 are shown in red on the YscU_C_ structure (2JLI.PDB). The YscU_CN_ and YscU_CC_ fragments are colored orange and gray, respectively. The two histidine residues (266 and 324) in the folded part of YscU_C_ are indicated. (**E**) Coomassie stained gels show Yop secretion under different pH conditions. (top panel) “pellet” indicates intracellular proteins; (middle panel) “supernatant” denotes secreted proteins. The YopJ protein (black box) was subjected to densitometric analysis for quantification of secretion levels (see Table 2). (bottom panel) The pH-dependency of YscU_CC_ secretion was visualized on immunoblots with anti-YscU_CC_ peptide antibodies.

To compare and quantify the Yop secretion efficiency in wild-type *Y. pseudotuberculosis* under different conditions, Coomassie stained Yop secretion profiles were subjected to densitometric analysis with Multi Gauge software (Fuji Film). The protein YopJ (boxed in Figure 4 and Figure 5E) was selected for quantitative analysis. Growth kinetics in media with different pH’s (Figure S4B) showed attenuation of bacterial growth directly linked to the observed Yop secretion efficiency.

a secretion efficiency was set to 100%;

b secretion efficiency at pH 7.5 was set to 100%.

### GST-pulldown Assay

We purified GST-YscU_C_-His_6_ and GST-A268F-His_6_ proteins in 2 steps, by combining GST- and Ni-NTA affinity chromatography. Purified proteins (80 µM) were incubated in 25 mM Tris, pH 7.4, 1 mM EDTA, and 150 mM NaCl at 37°C and 30°C. At different time points, 250 µl samples were taken, centrifuged to remove aggregates (15 min, 16 000×*g* at 4°C), and loaded on GST-SpinTrap™ columns (GE Healthcare). Columns were washed twice with 25 mM Tris, pH 7.4, 150 mM NaCl buffer, and eluted twice by adding 20 mM GSH solution, pH 8.0. Eluted samples were mixed with SDS-sample buffer and boiled. Proteins were subsequently separated and visualized with 4–12% Bis-Tris Gel SDS-PAGE (Invitrogen).

### Protein Purification of YscU_C_ Variants

All YscU_C_ constructs were cloned as GST fusion with a cleavage site for PreScission Protease between the GST domain and YscU_C_ (see supporting “Material and methods S2”). After transformation into *E. coli* BL21 (DE3) pLysS the protein synthesis was induced with IPTG and performed overnight at 30°C in LB medium containing carbenicillin and chloramphenicol. The bacterial cells were harvested by centrifugation at 5 000 rpm at 4°C and stored at −80°C until use. The protein purification of YscU_C_ variants was performed with an ÄKTA purifier system (GE Healthcare). The bacterial pellet was resuspended in 50 mM Tris pH 7.4 and 2 mM DTT, and cells were disrupted by sonication. The lysate was clarified by centrifugation at 15 000 rpm and 4°C, and the supernatant passed through a 0.45 µm syringe filter (Corning). The lysate, containing the soluble GST fusion protein, was loaded on a 5 mL GSTrap FF column (GE Healthcare) and eluted with 20 mM GSH solution at pH 8.0. Fractions with the fusion protein were pooled, dialyzed at 4°C against cleavage buffer (50 mM Tris pH 7.4, 150 mM NaCl, 1 mM EDTA, 1 mM DTT). The GST protein was cleaved from the target protein by adding PreScission Protease (GE Healthcare). To remove GST and non-cleaved GST-fusion protein the solution was passed through a Glutathione Sepharose 4B column. The flowthrough with YscU_C_, was subjected to cation exchange chromatography (5 ml SP Sepharose, GE Healthcare). All eluted fractions with YscU_C_ were pooled, concentrated with Amicon Ultra-15 Centrifugal Filter Units (Millipore, Billerica, MA), and polished with size-exclusion chromatography (HiPrep 26/60 Sephacryl S-100HR, GE Healthcare) in phosphate buffered saline at pH 7.4. Fractions with YscU_C_ were pooled stored as 100 µM stock at 20°C until use.

**Figure 6:**
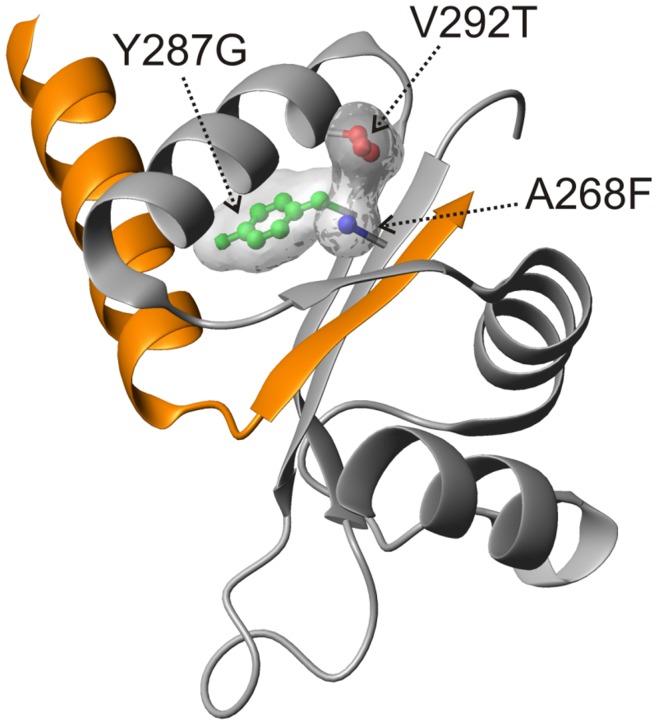
YscU_C_ suppressor mutations are buried in the structure. The spatial locations of single mutations in YscU_C_ that suppressed the non-secreting Δ*ysc*P phenotype *in vivo* are shown on the YscU_C_ structure of *Y. pestis* (2JLI.PDB). All positions are either fully or partially buried in the protein structure. YscU_CN_ and YscU_CC_ polypeptides are colored orange and gray, respectively.

### Analytical Size Exclusion Chromatography

Protein samples (YscU_CC_) were applied to a Superose 6 10/300 GL column (GE Healthcare) and size-exclusion chromatography (SEC) was performed with a flow rate of 0.5 ml/min in phosphate buffered saline at pH 7.4. Protein elution was followed by monitoring the UV absorption at 260 nm, 280 nm, and 220 nm. All samples with signal peaks at 280 nm were analyzed by SDS-PAGE. Prior to analytical SEC, the column was calibrated with a gel filtration protein standard (Bio-Rad).

**Figure 7:**
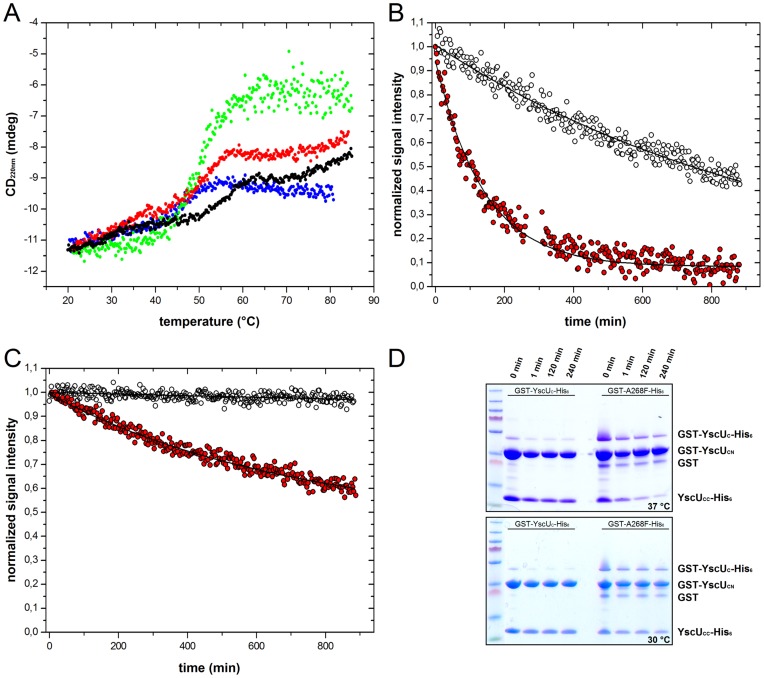
YscU_C_ suppressor mutant stabilities and dissociation kinetics. (**A**) Thermal induced unfolding of YscU_C_ and suppressor mutants. Dissociation temperatures (*T*_diss_) of single suppressor mutants at pH 7.4 were quantified with the CD signal at 220 nm and a scan rate of 1°C/min; YscU_C_ (black), A268F (blue), Y287G (green), and V292T (red). All suppressor mutants are destabilized compared to wild-type YscU_C_. *T*_diss_ values are summarized in Table 3. (**B**), (**C**) Dissociation kinetics quantified as dissociation life-times (τ_diss_) of YscU_C_ (black) and V292T (red) at pH 7.4 followed with NMR-spectroscopy at (B) 37°C and (C) 30°C, respectively. Primary NMR data for (B) is shown in Figure S6. Solid lines correspond to fits of the experimental data to single exponential decays. (**D**) Time dependent GST-pulldown experiments show the dissociation of wild-type YscU_C_ and the suppressor mutant A268F at 30°C and 37°C after varying incubation times. The suppressor mutant A268F displayed pronounced dissociation of YscU_CC_-His_6_ at 37°C and moderate dissociation at 30°C; wild-type YscU_C_ displayed no dissociation of YscU_CC_-His_6_ at 37°C or at 30°C over the observed time period. Note! Dissociation of YscU_CC_ is manifested as disappearance of YscU_CC_-His_6_ over time since the dissociation is irreversible and YscU_CC_-His_6_ cannot bind itself to the used resin.

**Table 3:** Dissociation temperatures and kinetics of wild-type YscU_C_ and the suppressor mutants V292T, Y287G, and A268F probed with NMR and CD spectroscopy.

Circular dichroism^a^	*T*_diss_ (°C)^b^	*τ*_diss_ at 37°C (min)
YscU_C_, wild-type	55.2±1.4	stable^c^
V292T	49.5±0.5	60.8±3.2
Y287G	48.9±0.3	138.4±10.5
A268F	44.8±0.5	70.1±4.8
		
NMR spectroscopy^d^	*τ*_diss_ at 30°C (min)	*τ*_diss_ at 37°C (min)
YscU_C_, wild-type	29914±3526	827±71
V292T	714±47	140±3

CD spectroscopy at 220 nm was performed at a scan rate of 1°C/min to determine the dissociation temperature (*T*_diss_) of YscU_C_ and suppressor mutants (Figure 7A). The kinetics of the dissociation process (*τ*_diss_) was monitored with CD spectroscopy at 220 nm and NMR spectroscopy at 37°C and 30°C. See also Figure 7B and 7C; Figure S5B.

a measured by following the CD signal at 220 nm;

b measured with a scan-rate of 1°C/min;

c dissociation was too slow to fit with a single exponential decay function;

d measured by following methyl group intensities in one dimensional ^1^H spectra.

### NMR Spectroscopy

NMR experiments were performed on a Bruker DRX 600 MHz spectrometer equipped with a 5-mm triple resonance z-gradient cryoprobe. Temperature calibration was conducted with a home-made probe, inserted into the sample compartment of the cryoprobe. The NMR samples contained unlabeled, ^15^N-labeled, or ^15^N/^13^C enriched protein in a buffer consisting of 10% ^2^H_2_0 (v/v), 50 mM NaCl, and 30 mM phosphate buffer at pH 7.4. Backbone YscU_C_ resonance assignments were accomplished with triple resonance experiments, HNCA [28], HNCOCA, HNCACB [29], and CBCACONH [28], supplemented with a ^15^N NOESY-HSQC experiment. Chemical shift perturbations were calculated according to: Δω = 0.2 · |Δ^15^N|+|Δ^1^H| (ppm).

The time series of one-dimensional ^1^H NMR spectra to probe dissociation were acquired with a pulse program from the Bruker library, which incorporated excitation sculpting for water suppression. For each spectrum, 64 scans were accumulated with a relaxation recovery delay of 2 s between scans. For each protein, time series were acquired at 30°C and 37°C. To quantify dissociation kinetics, we integrated the methyl group resonances in the 0.2 to 0.4 ppm spectral region. Each time course of the NMR signal was fit with a single exponential decay function of the form: I = I_0_ exp^(−t/τdiss)^+A, where τ_diss_ was the lifetime of the decay, and A was a baseline offset. NMR data was processed with NMRPipe [30] and visualized in ANSIG for Windows [31].

**Figure 8:**
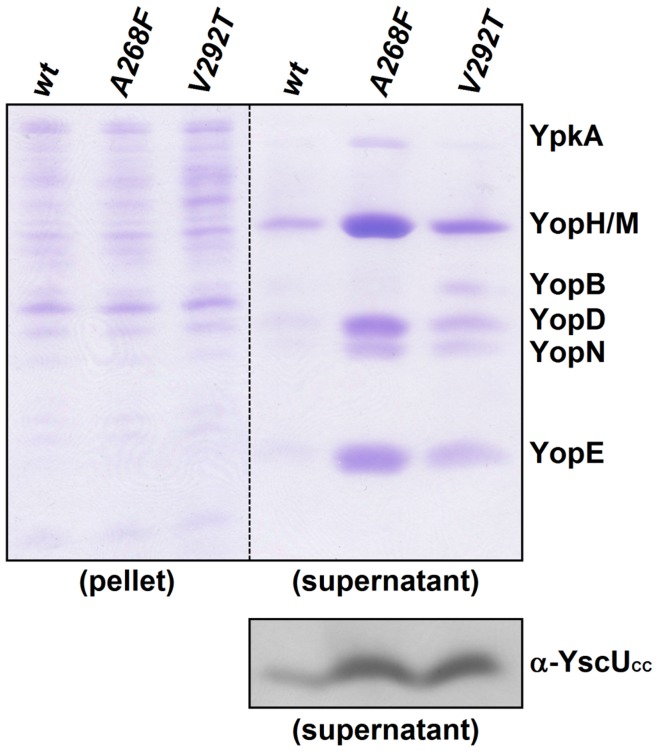
*Yersinia* strains with destabilized *ysc*U suppressor mutants secrete YscU_CC_ and Yops at lower temperatures (30°C) than wild-type. Coomassie stained analysis of Yop secretion in *Y. pseudotuberculosis* incubated at 30°C. Bacteria expressing either wild-type *ysc*U or one of the suppressor mutants, *A268F* or *V292T*. “pellet” indicates intracellular proteins; “supernatant” denotes secreted proteins. Secretion of YscU_CC_ was analyzed on immunoblots with anti-YscU_CC_ peptide antibodies. *Yersinia* harboring *ysc*U suppressor mutants showed strongly elevated secretion of Yops after cultivation at 30°C.

### Circular Dichroism

Circular dichroism (CD) spectra were recorded on a Jasco J-810 spectropolarimeter, equipped with a Peltier element for temperature control and a 0.1 cm quartz cuvette. The proteins were measured at 10 µM in a buffer of 10-fold diluted phosphate buffered saline at pH 7.4. For all experiments with calcium, different buffers were used (phosphate, MOPS, and Pipes) to exclude possible calcium precipitation effects. Thermal dissociation experiments were performed by monitoring the CD signal at 220 nm as a function of temperature. All thermal profiles were acquired in the interval of 20°C to 90°C. The thermal scan rate was varied from 0.5 to 2°C/min, without any significant change in protein behavior; we selected 1°C/min as the standard condition in this study for CD spectroscopy-based temperature perturbation experiments. The inflection point for dissociation, *T*_diss_, was quantified by fitting thermal curves with a two-state equation [32]. Calcium binding affinity was quantified by fitting a one-site binding model to the CD data.

## Results

### Dissociation of YscU_CC_ from YscU_CN_

We recently published results showing that specific *ysc*U mutants (*N263A* and *P264A*), defective in autoproteolysis, were impaired in their ability to secrete Yops into the culture supernatant at wild-type levels [16]. These mutations strongly reduced the autoproteolytic activity of the YscU protein. Especially the *ysc*U mutant *P264A* was severely suppressed in autoproteolysis leading to an almost complete inhibition of Yop secretion [16]. These results and earlier findings showing that deletion of the autoproteolytic cleavage motif NPTH leads to a complete loss of Yop secretion indicated that the cleavage of YscU is required for Yop secretion [18]. Here, we decided to study YscU in more detail. Because YscU is an integral inner-membrane protein, we produced a polypeptide that comprised the cytosolic segment of YscU including the motif for autoproteolytic cleavage (YscU_C_; Figure 1A and Figure 1B) for *in vitro* studies. For detailed investigation of conformational changes in YscU_C_ we have used the spectroscopic methods nuclear magnetic resonance (NMR) and circular dichroism (CD).

It was previously proposed that the YscU_CC_ polypeptide dissociates from the remaining, membrane-anchored segment of YscU to allow Yop secretion [16]. To test this hypothesis, we developed biophysical NMR and CD based protocols for the quantification of YscU_CC_ dissociation from YscU_CN_ *in vitro*. The high quality, ^1^H-^15^N HSQC spectrum of YscU_C_ at 20°C (Figure 2A, blue contours) and the chemical shift dispersion showed that YscU_C_ was a folded protein under the experimental conditions. We assigned 91% of the non-proline backbone resonances in our protein construct that contained residues 211–354. In the YscU_C_ crystal structure (2JLI.PDB) the N-terminal residues 241–255 are in a helical conformation. The helix protrudes into solution and the first residue that makes contact with the remainder of the protein is residue number 250. In solution the first helical residue that we could identify based on NOE contacts was residue 251, and the assigned preceding residues are adopting an unstructured and flexible conformation as inferred from high signal intensities and narrow chemical shift dispersion. After incubation at 60°C for 10 min, the NMR spectrum of YscU_C_ at 20°C showed a dramatic perturbation (Figure 2A, red contours); only resonances that corresponded to the YscU_CN_ polypeptide were visible. The narrow chemical shift dispersion and high peak intensities of the YscU_CN_ resonances showed that the polypeptide adopted an unfolded conformation also after dissociation from YscU_CC_. The absence of signals that corresponded to the YscU_CC_ polypeptide was due to the formation of aggregated particles too large for detection with NMR spectroscopy (see supporting material, “Results S1”).

The NMR experiments showed that YscU_CC_ dissociation from YscU_CN_ was triggered by subjecting YscU_C_ to thermal perturbation, and that dissociation was an irreversible process. The irreversibility provided a tool for quantifying dissociation kinetics (discussed below). To obtain an accurate value of the dissociation temperature (*T*_diss_), we observed YscU_C_ dissociation by subjecting the protein to a thermal cycle, and we followed this event with CD at 220 nm. The CD signal at 220 nm contains contributions from both alpha helical and β-strand secondary structures [33]. Thus, because YscU_C_ contains, both alpha helices and β-strands, 220 nm was a suitable wavelength for following changes in the YscU_C_ structure (Figure 1B). The thermogram of YscU_C_ (Figure 2B) was composed of two distinct transitions (55°C and 77°C), and the overall thermal response was not reversible, as the CD signal did not reach its initial value after a complete thermal cycle (see supporting material, “Results S2”). The transition at 55°C corresponded to the dissociation of YscU_C_; this was in good agreement with the NMR results that showed an upper limit of *T*_diss_ equal to 60°C. The high temperature transition (77°C) was reversible, but with distinct signs of hysteresis (Figure S1). Because this transition was not relevant in the context of dissociation, we did not study it further. Of note, because dissociation is an irreversible process, *T*_diss_ is scan-rate dependent. Therefore, all CD spectroscopy-based temperature perturbation experiments were conducted at a fixed scan rate of 1°C/min.

To address the questions whether the dissociation of YscU_C_ might have biological relevance we subjected the non-cleavable YscU_C_ mutant P264A to a thermal denaturation. The thermal signature of P264A was dramatically perturbed compared to the wild-type, and the thermogram displayed one irreversible transition at 55°C (Figure 2C). To investigate the biological relevance further, we asked whether calcium might affect the YscU_C_ dissociation *in vitro*. The thermogram in the presence of calcium was remarkably similar to that of the non-cleavable variant P264A in the absence of calcium (Figure 3A). Hence, calcium mimicked the effect of a mutation that suppressed the YscU_C_ auto-processing activity. P264A contained one polypeptide chain that could not dissociate; thus, the data suggested that calcium prevented dissociation of YscU_CC_ from the YscU_CN_ polypeptide.

Next we investigated the ability of YscU_C_ to bind calcium *in vitro* and compared it to the calcium concentration needed to block the Yop secretion *in vivo*. The CD signal at 220 nm indicated the YscU_C_ binds calcium with a dissociation constant (*K_d_*) of 800 µM, assuming a one-site binding model (Figure 3B). To benchmark the *K_d_* value of calcium against the calcium concentration required for inhibition of the *Yersinia* T3SS *in vivo*, we analyzed Yop secretion and expression at different calcium levels in the growth medium. The T3SS was down regulated at calcium concentrations between 0.5 to 1 mM (Figure 3C). Hence, the *in vitro K_d_* value for calcium interaction with YscU_C_ (800 µM) was well in the concentration interval that inhibited Yop secretion *in vivo*. Comparative analysis with different divalent cations revealed no exclusive specificity of YscU_C_ towards calcium. Different alkaline earth metals, Mg^2+^, Ca^2+^, Sr^2+^ and Ba^2+^ showed comparable effects *in vitro* on YscU_C_ interaction and dissociation (Table 1). It was not surprising that Ba^2+^ and Sr^2+^ showed a similar effect as Ca^2+^ since these ions have been shown to effect Yop secretion similarly to Ca^2+^ [34]. On the other hand Mg^2+^ has no effect on Yop secretion suggesting that the *in vivo* regulation of calcium controlled secretion is dependent on additional factors. This promiscuous metal binding property of YscU_C_ is consistent with the absence of any known calcium binding motif in YscU_C_. In accordance we observed with NMR spectroscopy that calcium binding is mediated through a large set of residues confined to the YscU_CC_ polypeptide (Figure S2).

Nevertheless the *in vitro* data clearly showed that YscU_C_ was poised for dissociation into YscU_CN_ and YscU_CC_ fragments, and that this event can be inhibited by calcium and other divalent cations. Thus we wondered whether the dissociation of YscU_C_ could be monitored directly in *Yersinia*, and whether it can be linked to T3SS regulated Yop secretion. To test this idea, we first probed for the presence of YscU_CC_ in the culture supernatants after incubating the wild-type *Y. pseudotuberculosis* strain at 37°C in the absence or presence of 2.5 mM calcium. Remarkably, YscU_CC_ was found in the culture supernatant from the calcium depleted cultures, and no YscU_CC_ was found in cultures with 2.5 mM calcium (Figure 4). To explore this finding further, the gene for the YscU_CC_ polypeptide (amino acids 264 to 354 of YscU) was cloned into the pBAD vector under the control of an inducible *ara*C promoter. This allowed the *in trans* overexpression of *yscU_CC_* in *Y. pseudotuberculosis*. After promoter induction, the levels of secreted YscU_CC_ were analyzed in cultures grown with or without calcium. We found that induction of *yscU_CC_* expression caused increased secretion of YscU_CC_ into the culture supernatant when compared to the wild-type levels secreted by a strain that contained the control vector. Further, secretion of YscU_CC_ was blocked in the presence of 2.5 mM calcium in the medium. To investigate the requirement of a functional T3SS for YscU_CC_ secretion we analyzed the secretion behavior of a *Y. pseudotuberculosis* Δ*ysc*C null mutant. In absence of YscC no secretion of either Yops or YscU_CC_ was observed (Figure 4) showing that secretion of YscU_CC_ is dependent on a functional T3SS. To link our *in vitro* findings of YscU_C_ and calcium directly to *in vivo* events we analyzed the secretion behavior of a Δ*yop*N mutant that has lost its calcium regulation and secretes Yops in presence and absence of calcium in similar amounts [35]. It was found that the Δ*yop*N mutant secreted YscU_CC_ independently of the calcium concentration. Thus, YscU_CC_ was secreted from the Δ*yop*N mutant in the presence of calcium showing a similar secretion profile as the Yop substrates (Figure 4).

In conclusion, YscU_CC_ showed a secretion pattern similar to that of Yops. This suggested that YscU_CC_ constituted a novel substrate of the T3SS in *Yersinia*. Furthermore, the fact that YscU_CC_ was secreted in the wild-type strain demonstrated that YscU_CC_ was able to dissociate from the remaining membrane bound part of YscU *in vivo*.

### pH-dependencies of YscU_CC_ Dissociation/secretion and Yop Secretion

Unpublished observations from our laboratory indicated that Yop secretion, but not bacterial growth, was influenced by the pH of the growth medium. Further it has been shown that autoproteolysis of YscU_C_ was pH dependent [15]. Here, we addressed the question of whether dissociation of YscU_C_ was also pH dependent. First, we subjected cleaved YscU_C_ to a thermal cycle at pH 6.0 (instead of pH 7.4) by monitoring the thermal signature with CD spectroscopy at 220 nm (Figure 5A). When the resulting thermogram was super-imposed on the thermograms of the wild-type YscU_C_ at pH 7.4 in presence of 2.5 mM calcium and the non-cleavable mutant P264A in the absence of calcium, they were virtually identical. From this observation, we concluded that YscU_C_ dissociation was prevented by low pH. YscU_C_ contains two histidine residues (positions 266 and 324) in the YscU_CC_ fragment that may explain the observed pH-dependency. By monitoring the CD signal at 220 nm as a function of pH, we identified one ionization event in the pH interval of 6.0 to 7.4, with a pKa value of around 6.3 (Figure 5B), and one ionization event around pH 8.0. NMR analyses revealed that both histidines were protonated in response to a pH drop from 7.4 to 6.0 (Figure 5C, 5D). This indicated that the protonation of histidines 266 and 324 was responsible for the observed differences in thermally induced dissociation of YscU_C_ at pH 6.0 and 7.4. This observation was interesting, because all known YscU orthologs in other T3SSs harbor a conserved histidine residue at the position that corresponds to amino acid 266 in the N↑PTH motif. To further dissect the relevance of the two histidines we replaced histidine 324 with alanine (H324A) and studied the *in vitro* response of this mutant to both thermal- and pH perturbations. Since it has been shown that mutation of histidine 266 leads to an *ysc*U mutant affected in autoproteolysis this position is not suitable for an alanine replacement [36]. The H324A variant showed similar dissociation behavior *in vitro* but with reduced thermal stability at pH 7.4 and pH 6.0 compared to wild-type (Figure S3A and S3B). The pH-dependency of the CD-signal at 220 nm of the histidine mutant H324A showed a distinct difference compared to the wild-type protein (Figure S3C). Whereas the wild-type protein displayed two ionization events (at pH 6.3 and 8.0) the mutant only displayed the ionization event at pH 6.0. Hence, the pKa of the N↑PTH histidine is around 6, and protonation/deprotonation of this histidine is likely responsible for the difference in thermally induced dissociation at pH 6.0 and 7.4. Next, we wondered whether these biophysical observations reflected biological effects *in vivo*. To investigate the pH-dependency of Yop secretion, we cultivated wild-type *Y. pseudotuberculosis* in Hepes buffered media at pH values between 6.0 and 7.5 in calcium depleted media and monitored the secretion of YscU_CC_ and Yops (Figure 5E and Table 2). Both Yop and YscU_CC_ secretion was maximal at pH levels between 7.0 and 7.5. Secretion gradually decreased when pH was lowered, and at pH 6.0, we observed a pronounced inhibitory effect on the secretion of YscU_CC_ as well as the Yops (albeit not as strong as the inhibition by calcium). Importantly, bacterial growth was not affected by changing the pH of the growth medium; thus, perturbations of external pH values did not cause any general effects on bacterial proliferation (Figure S4). These *in vivo* and *in vitro* results suggested that the pH-dependent secretion of the secretion of Yops and YscU_CC_ was a consequence of the molecular behavior of YscU; i.e., the dissociation and secretion of YscU_CC_ was a prerequisite for maximal secretion of Yop substrates into the culture medium.

### Yops are Secreted at Lower Temperatures in Destabilized YscU_CC_ Variants Compared to Wild-type

The results described above suggested that dissociation of YscU_CC_ from the remainder of YscU, followed by secretion of the YscU_CC_ polypeptide, was important for Yop secretion. We and other groups previously showed that single amino acid substitutions in YscU_C_ could suppress the Yop secretion-deficient phenotype of the *Y. pseudotuberculosis* Δ*ysc*P mutant [17], [21], [37]. Identification of such suppressor mutations is generally considered as strong genetic evidence for protein-protein interactions. Thus, second-site suppressor mutations are expected to be localized at protein surfaces that are prime positions for protein-protein interactions. In sharp contrast, all amino acid substitutions on the YscU_CC_ polypeptide were either fully or partially buried in the protein structure (Figure 6). Consequently, the underlying mechanism of these mutations must be more complex than a direct protein-protein interaction with YscP. Mutations at buried positions generally act to destabilize proteins; therefore, we reasoned that the altered secretion behavior of the suppressor mutants might be attributed to perturbed stabilities (*T*_diss_) and dissociation rates (*k*_diss_ = 1/τ_diss_) compared to wild-type YscU_C_.

To test this notion, we monitored the dissociation kinetics of YscU_C_ suppressor mutants A268F, Y287G, V292T and wild-type YscU_C_ with CD and NMR spectroscopy *in vitro*. The resulting dissociation kinetics are reported as dissociation lifetimes (τ_diss_), or the reciprocal of the dissociation rate ( = 1/*k*_diss_). Because Yop secretion is triggered by a temperature shift from 26°C to 37°C [3], [4], [6], we initially performed the assays at 37°C. We found that all YscU_C_ mutants were folded (Figure S5A) and displayed decreased thermal stabilities compared to wild-type YscU_C_, evident from the reduced dissociation temperatures (Figure 7A and Table 3). We quantified the dissociation kinetics with both CD and NMR spectroscopy for the YscU_C_ variant V292T; with CD, we detected changes in ellipticity at 220 nm that accompanied dissociation (Figure S5B); with NMR, we detected the loss of resonance intensities for residues in the YscU_CC_ fragment (Figure S6). The time-dependent signals were well described by first order processes; accordingly, all kinetic traces could be fit accurately with single exponential decay functions. Both CD and NMR results indicated that wild-type YscU_C_ displayed very slow dissociation kinetics at 37°C in the observed time frame; in comparison, the YscU_C_ variant V292T displayed a significantly enhanced rate of dissociation (Figure 7B, Figure S6A, S6B). It should be noted that the observed variations in the dissociation lifetimes (*τ*_diss_) for the suppressor mutants are dependent on the method used for quantification (Table 3). For instance, *τ*_diss_ for the V292T variant was 61 min and 140 min, based on CD and NMR, respectively. This discrepancy could be attributed to differences in sensitivity; the CD signal was directly sensitive to the dissociation process, but NMR required both dissociation and aggregation for a reduction in signal intensity. Hence, both dissociation and aggregation were slow processes that occurred at similar time scales *in vitro*. After one hour at 37°C, a significant fraction of the V292T variant displayed dissociated species (65% and 38% from CD and NMR, respectively), but wild-type YscU_C_ remained intact under the same conditions.

V292T displayed a significantly reduced thermal stability compared to wild-type YscU_C_; therefore, we also performed NMR-based kinetic experiments at 30°C (Figure 7C). At this temperature, wild-type YscU_C_ was stable over the entire experiment (860 min), but the V292T variant dissociated at a rate equal to the rate for wild-type YscU_C_ at 37°C, within experimental error (Table 3). We confirmed these results with the A268F suppressor mutant. Further we used a GST pull down assay, and the dissociation of GST-YscU_C_-His_6_ respectively GST-A268F-His_6_ was monitored at 30°C and 37°C. Indeed, GST-YscU_C_-His_6_ was completely stable at both temperatures, but GST-A268F-His_6_ dissociated to a significant extent over time, visible in the decrease of the YscU_CC_-His_6_ content at both 30°C and 37°C (Figure 7D).

Because the dissociation of YscU_C_ is required for Yop secretion *in vivo*, we expected suppressor mutant strains to secrete Yops at lower temperatures compared to the wild-type strain. To test this hypothesis, we probed for the presence of secreted Yops and YscU_CC_ in the culture supernatant in strains that carried either *ysc*U or *ysc*U suppressor mutants *A268F* or *V292T* after a temperature shift from 26°C to 30°C. Both the *A268F* and *V292T* mutant strain secreted Yops and YscU_CC_ already at 30°C; in contrast, the wild-type strain showed almost no secretion at this temperature (Figure 8). Probing for LcrV and YscI as early substrates in T3SS confirmed the direct link between secretion of YscU_CC_ and T3SS substrates (Figure S7B). Despite the increased secretion of Yops at reduced temperatures, the pH regulation was still active in these *Yersinia* mutants. *A268F* and *V292T* revealed the same secretion pattern like the wild-type when grown in media with different pH (Figure 5E and Figure S7A). Importantly, all strains secreted Yops in a calcium-regulated manner; this showed that the suppressor mutants retained calcium sensing capability (data not shown).

Our results showed a strong link between *in vivo* and *in vitro* results for dissociation of the YscU_CC_ polypeptide from the remainder of the protein. This demonstrated that not only autoproteolytic cleavage but also dissociation and secretion of YscU_CC_ is a key step in the regulation of Yop secretion.

## Discussion

The YscU protein of *Yersinia pseudotuberculosis* and orthologs in other bacteria display autoproteolytic activity, with cleavage at a conserved N↑PTH motif. It is reasonable to assume that a strictly conserved autoproteolytic activity in a protein is linked to a specific function in the organism. Auto-processing has been observed in other proteins; e.g., in the SEA domain of the membrane-bound MUC1 protein, the processing occurs at a conserved GD↑PH site. It has been suggested that this cleavage introduces a molecular-mechanical fracture that protects epithelial cells from rupture [38]. It was previously postulated that autoproteolysis of YscU exposes a new binding surface to other T3SS proteins by changing the charge distribution at the cleavage site [39]. Here, we propose an alternative model, where dissociation of YscU_CC_ from the membrane anchored segment of YscU, followed by YscU_CC_ secretion via the T3SS, plays a central role in the substrate specificity switch [21].

We showed that T3SS mediated Yop secretion correlated with the secretion of YscU_CC_ and presumed a fully functional T3SS. Secretion of YscU_CC_ required autocatalytic cleavage of the cytosolic domain (YscU_C_) of the inner membrane protein YscU that was linked in earlier studies to be one key regulator in the substrate specificity switch of T3SS mediated Yop secretion [17]. *In vitro* analysis with recombinantly produced YscU_C_ confirmed the dissociation capacity of the protein and revealed potential regulating factors, like divalent ions (i.e. calcium), pH and temperature. We showed that T3SS mediated Yop secretion was strongly affected by 0.5 to 1 mM calcium and pH values below 6.5. The calcium concentration had an “all or nothing” effect on Yop secretion. In contrast, the inhibitory effect of low pH values was less pronounced. Surprisingly Ca^2+^-ions bound to YscU_C_ with a *K_d_* of 800 µM *in vitro*; notably, this value was consistent with the threshold value for calcium dependent down-regulation of Yop secretion *in vivo*. Additional *in vitro* analysis including different divalent cations Mg^2+^, Sr^2+^ and Ba^2+^ indicated that the alkaline earth metal ions interacted and stabilized YscU_CC_ binding to about the same extent. These results indicated that the calcium regulation of the *Yersinia* T3SS *in vivo* is complex and cannot be explained only on basis of the YscU_C_ calcium interaction. The N↑PTH histidine, is unfortunately not suitable for mutations to other amino acids since it affects the autoproteolytic activity [36]. However experiments where histidine 324 was replaced with alanine indicated that, overall, the N↑PTH histidine is responsible for the pH-dependency of YscU_C_ dissociation. Thus these findings suggest that pH is also an extracellular queue regulating the *Yersinia* T3SS.

It is known that the YscP protein plays an important role in the regulation of the substrate specificity switch [17]. We recently proposed that YscP activity may stimulate displacement of YscU_CC_ from the remaining YscU part, and that this activity was triggered by target cell contact or calcium depletion. We further suggested that, in *ysc*U suppressor mutants, the point mutations in YscU_CC_ induced a perturbation in the YscU structure, which destabilized the interaction between YscU_CC_ and the remaining membrane anchored part of YscU [16]. This was suggested because the whole *ysc*P gene was deleted; thus, it was likely that the YscU_CC_ mutations caused a gain of function. In the present study, we confirmed this hypothesis by showing that destabilization of the suppressor mutants led to premature YscU_CC_ dissociation, which then induced Yop secretion. Strains that carried the suppressor mutations secreted Yops at 30°C in calcium-depleted medium. This finding contrasted with findings in the wild-type strain, which showed almost no Yop secretion at 30°C. Nevertheless, all suppressor mutants retained the wild-type calcium regulation of Yop secretion indicating that calcium exhibits a stabilizing effect on the interaction between YscU_CC_ and the remainder of YscU.

Surprisingly, we found that YscU_CC_ was also secreted via the T3SS; this was remarkable, given that YscU is an inner membrane protein [40]. Our results favor a model where YscU orchestrates obstruction of the T3SS secretion channel. This block is thus, relieved through YscU_CC_ secretion in calcium depleted and in pH ≥6.5 conditions. In support for this model is the observation that *ysc*U mutants impeded for autoproteolysis and subsequent secretion of YscU_CC_ (mutations within the NPTH motif) are unable to secrete the Yop-proteins [18]. Further, these mutants secrete elevated amounts of the early substrate YscF showing that the T3SS is active in these mutants and allows secretion of early but not late substrates [16]. The model explains why these mutants are also defective in Yop secretion. Further support for this model was our finding that the “Ca^2+^-blind” *yop*N mutant, unable to respond to extracellular calcium, secreted YscU_CC_ as well as Yops in Ca^2+^ containing conditions indicating that YscU_CC_ secretion is tightly coupled to Yop secretion. Thus, YopN is essential for the calcium response in *Yersinia* and in addition our results suggest that YopN has a role in the Ca^2+^ response *in vivo* leading to stabilization of YscU. Moreover, Mg^2+^ blocks YscU_CC_ displacement *in vitro* similarly to Ca^2+^ but in contrast the pathogen secretes Yops *in vivo* at high concentrations of Mg^2+^, indicating that Mg^2+^ is not interacting with YscU_C_ during *in vivo* conditions. Thus it is possible that YopN discriminates between the ions, allowing transport/uptake of Ca^2+^ but not Mg^2+^ during growth at 37°C *in vivo*. YopN has also been shown to be surface located when *Yersinia* is grown at 37°C in presence of calcium which agrees with its putative role as calcium scavenger [35].

This study has provided a new handle for investigating the function of YscU/FlhB proteins in other bacteria. Given the conservation within this family of proteins, it is likely that they also exhibit regulatory roles that involve secretion of the processed C-terminus of the protein. However, depending on life style, the actual triggering signal may differ among different pathogens. For example, *Salmonella* invasion is controlled by environmental pH, low oxygen, and acetate [41]. Although different intracellular regulatory pathways have been linked to these signals, virtually nothing is known about signal reception and interpretation by the pathogen. Based on the results presented here, it would not be surprising to find that some signals stimulated secretion of the *Salmonella* YscU_CC_ homolog, SpaS_CC_.

## Supporting Information

Figure S1 **Reversibility of the high temperature (77°C) transition of YscU_C_.** Thermal up- and down-scans of 10 µM YscU_C_ were monitored with CD spectroscopy at 220 nm. YscU_C_ was subjected to two sequential thermal cycles (one cycle: 20°C to 95°C and then back to 20°C). The color coding indicates: first up-scan (black), first down-scan (open gray), second up-scan (open blue) and second down-scan (open red). After initial loss of secondary structure, the high temperature transition is reversible.
(TIF)

Figure S2 **Calcium induced chemical shift perturbations in YscU_C_.** (**A**) Chemical shift differences were monitored with two-dimensional ^1^H-^15^N HSQC NMR spectra of YscU_C_ before and after saturation with calcium. Chemical shift differences are plotted against the primary sequence. The blue line indicates the threshold value (0.06 ppm) used in (B) to highlight amino acid residues affected by addition of calcium. Residues responding to calcium are confined to the YscU_CC_ fragment. (**B**) Structural distributions of residues that show significant chemical shift perturbations in response to calcium binding are shown in red on the YscU_C_ structure (2JLI.PDB). YscU_CN_ and YscU_CC_ fragments are colored orange and gray, respectively.
(TIF)

Figure S3 **Biophysical analysis of the YscU_C_ variant H324A.** (**A**) Thermal up- and down-scans of H324A at pH 7.4 monitored with CD spectroscopy at 220 nm in the absence of calcium. (**B**) Thermal up- and down-scans of H324A at pH 6.0 monitored with CD spectroscopy at 220 nm in the absence of calcium. Up- and down scans of H324A are shown in red and blue circles, respectively. (**C**) The pH-dependency of the CD-signal at 220 nm for H324A.
(TIF)

Figure S4 ***Y. pseudotuberculosis*** **growth kinetics were pH independent between pH 6.0 and pH 7.5.** Bacterial growth of wild-type *Y. pseudotuberculosis* was analyzed by monitoring the optical density (OD_600_) under (**A**) calcium-supplemented and (**B**) calcium-depleted conditions. Bacteria were cultivated 2 h at 26°C, then 3 h at 37°C. Samples were taken every hour to monitor the growth based on the measured OD_600_. No significant differences in growth kinetics were observed within the monitored pH-interval of 6.0 to 7.5.
(TIF)

Figure S5 **Secondary structure analysis and dissociation kinetics of YscU_C_ suppressor mutants monitored with CD spectroscopy.** (**A**) Far-UV CD spectra of 10 µM wild-type YscU_C_ (black) and the suppressor mutants A268F (blue), Y287G (green), V292T (red). The similarity in the shape of the CD signals indicates that all YscU_C_ variants have similar secondary structure. (**B**) Dissociation kinetics of YscU_C_ (black) and suppressor mutants A268F (blue), Y287G (green), and V292T (red) were monitored with CD spectroscopy at 220 nm and 37°C. The solid lines represent the best fit of a single exponential decay function to determine *τ*_diss_. Dissociation kinetics are summarized in Table 3. Note that the data points at time = zero have been normalized for clarity.
(TIF)

Figure S6 **Primary NMR data used to quantify dissociation kinetics of wild-type YscU_C_ and V292T at 37°C.** Shown are expansions of methyl resonances from one-dimensional ^1^H spectra at various time points for (**A**) wild-type YscU_C_ and (**B**) the V292T mutant (see Figure 7B and 7C).
(TIF)

Figure S7 **Secretion analysis of** ***ysc*** **U suppressor mutants** ***A268F*** **and** ***V292T*** **.** (**A**) Secretion analysis of *A268F* and *V292T* after cultivation in Hepes-buffered LB at different pH values. Yop secretion was induced by calcium depletion and a temperature shift from 26°C to 37°C. *A268F* and *V292T* showed elevated Yop secretion at pH≥6.5 and strong inhibition at pH 6.0 (**B**) Secretion analysis of *A268F* and *V292T* at pH 7.5 and 30°C compared to wild-type. The T3SS was induced by calcium depletion and a temperature shift to 30°C. *A268F* and *V292T* showed a strongly elevation of Yop secretion. Coomassie stained gels demonstrate secreted Yops. “pellet” indicates intracellular proteins; “supernatant” denotes secreted proteins. The secretion of YscI and LcrV was visualized on immunoblots with anti-YscI and anti-LcrV antibodies.
(TIF)

Figure S8 **Size exclusion chromatography-based estimation of the YscU_CC_ aggregate size.** Chromatogram of analytical size exclusion chromatography of purified YscU_CC_. YscU_CC_ eluted in the void volume of the SEC column.
(TIF)

Figure S9 **CD-based analysis of YscU_CC_ produced by thermal stimulation or by recombinant protein production.** Comparison of YscU_C_ CD spectra at 20°C after one completed thermal cycle to 95°C. Filled circles show the YscU_CC_ produced by thermal stimulation and open circles show the purified YscU_CC_ fragment, produced *in vitro* by recombinant protein production. The similarity of the spectra shows that the residual CD signal of YscU_C_ after one thermal cycle is dominated by the YscU_CC_ fragment.
(TIF)

Table S1 **Bacterial strains and plasmids used in this study.**
(RTF)

Table S2 **Primers used in this study.**
(RTF)

Materials and Methods S1 **Procedure for cloning** ***yscU*** **_CC_ into pBADmyc His B.**
(RTF)

Materials and Methods S2 **Cloning procedure for GST fusion proteins.**
(RTF)

Results S1 **YscU_CC_ aggregated after dissociation from YscU_CN._** Analytical size exclusion chromatography was used to show that YscU_CC_ aggregates after dissociation from the YscU_CN_ polypeptide.
(RTF)

Results S2 **Persistent secondary structure in aggregated YscU_CC_ from CD spectroscopy.** CD spectroscopy on thermally and recombinantly produced YscU_CC_ aggregates was used to show that YscU_CC_ contains elements of secondary structure in the aggregated state.
(RTF)
