# Supplementary figures and images for: Autoproteolysis and Intramolecular Dissociation of Yersinia YscU Precedes Secretion of Its C-Terminal Polypeptide YscUCC
Source: PLoS One. 2012 Nov 21;7(11):e49349. doi: 10.1371/journal.pone.0049349 (PMC3504009; doi:10.1371/journal.pone.0049349)

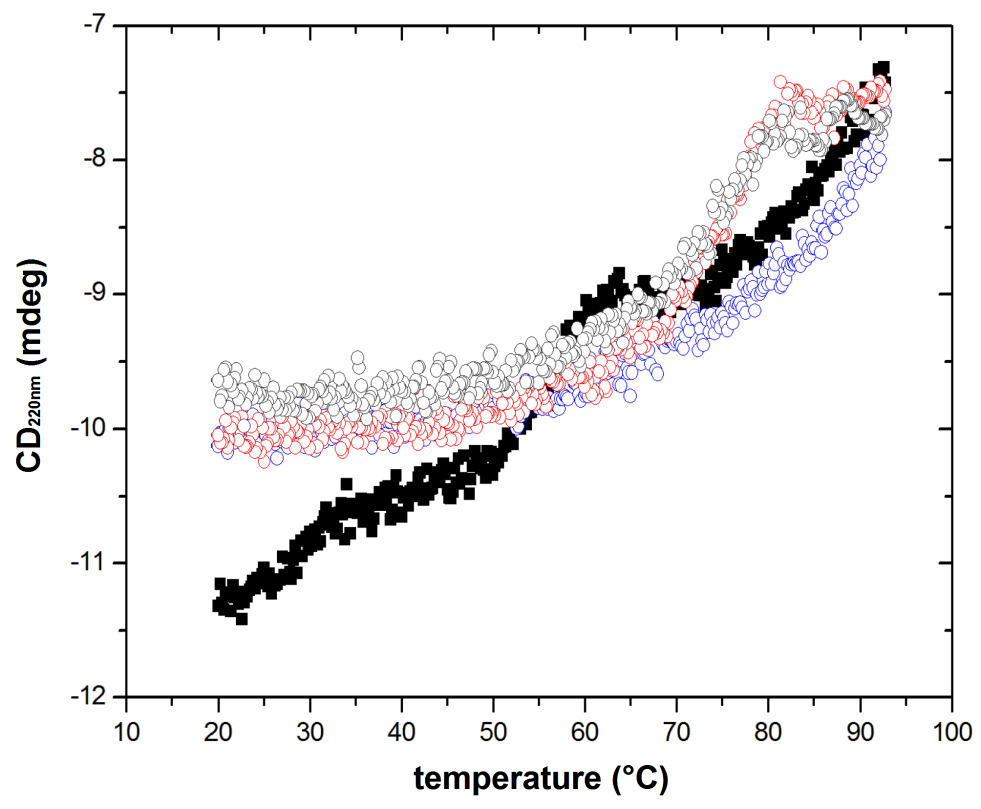

Supplement: Figure S1 — Reversibility of the high temperature (77°C) transition of YscUC. Thermal up- and down-scans of 10 µM YscUC were monitored with CD spectroscopy at 220 nm. YscUC was subjected to two sequential thermal cycles (one cycle: 20°C to 95°C and then back to 20°C). The color coding indicates: first up-scan (black), first down-scan (open gray), second up-scan (open blue) and second down-scan (open red). After initial loss of secondary structure, the high temperature transition is reversible. (TIF) [file pone.0049349.s001.tif]

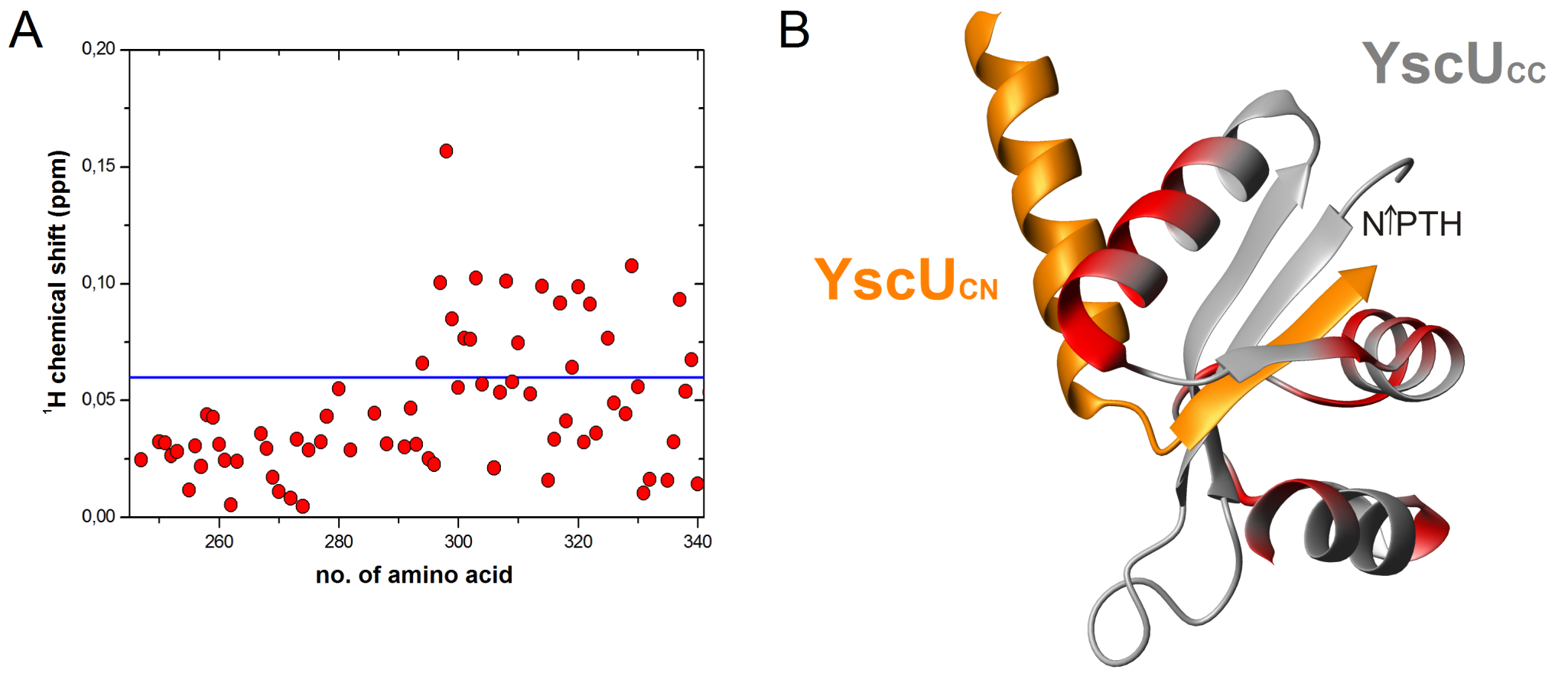

Supplement: Figure S2 — Calcium induced chemical shift perturbations in YscUC. (A) Chemical shift differences were monitored with two-dimensional 1H-15N HSQC NMR spectra of YscUC before and after saturation with calcium. Chemical shift differences are plotted against the primary sequence. The blue line indicates the threshold value (0.06 ppm) used in (B) to highlight amino acid residues affected by addition of calcium. Residues responding to calcium are confined to the YscUCC fragment. (B) Structural distributions of residues that show significant chemical shift perturbations in response to calcium binding are shown in red on the YscUC structure (2JLI.PDB). YscUCN and YscUCC fragments are colored orange and gray, respectively. (TIF) [file pone.0049349.s002.tif]

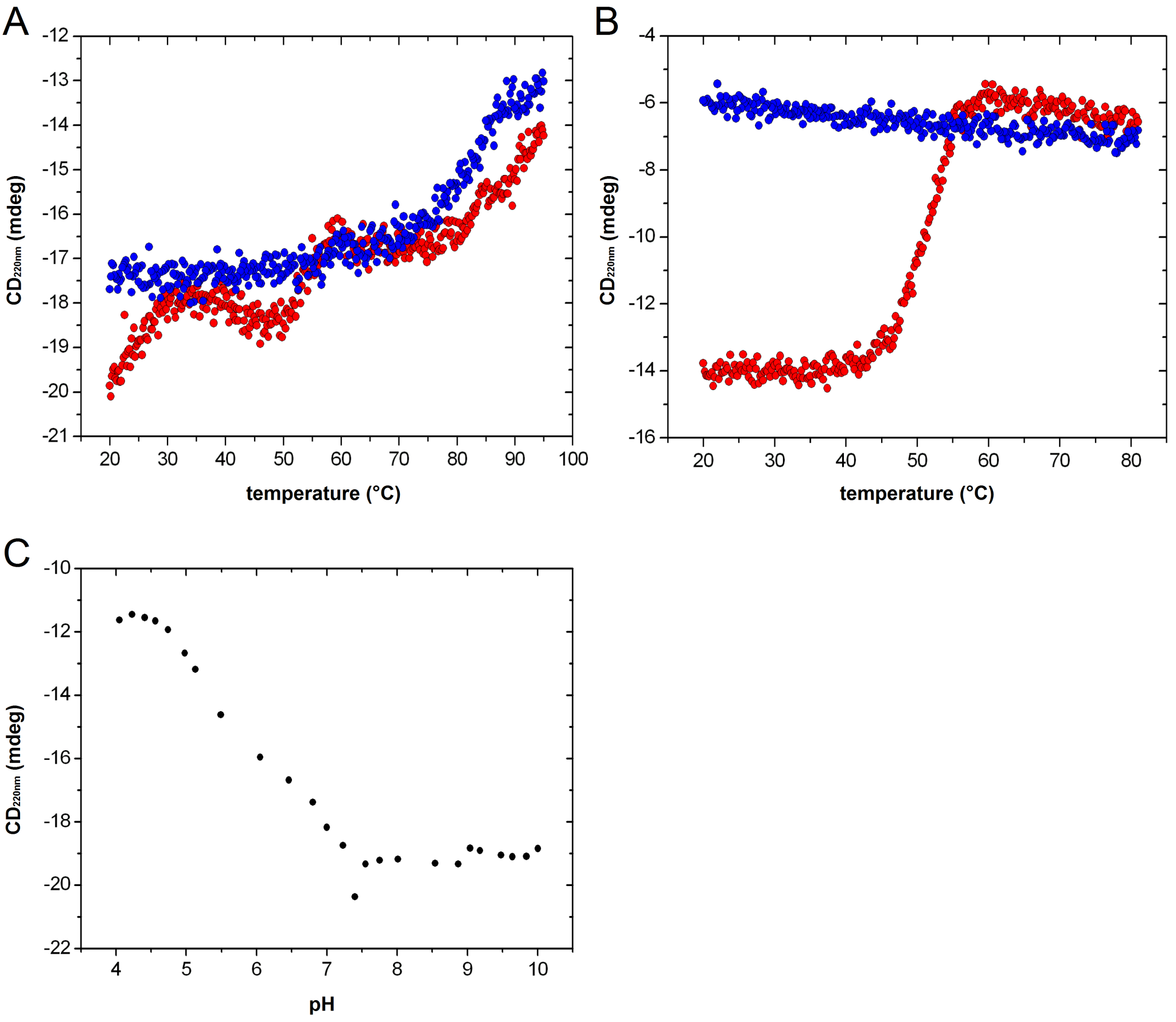

Supplement: Figure S3 — Biophysical analysis of the YscUC variant H324A. (A) Thermal up- and down-scans of H324A at pH 7.4 monitored with CD spectroscopy at 220 nm in the absence of calcium. (B) Thermal up- and down-scans of H324A at pH 6.0 monitored with CD spectroscopy at 220 nm in the absence of calcium. Up- and down scans of H324A are shown in red and blue circles, respectively. (C) The pH-dependency of the CD-signal at 220 nm for H324A. (TIF) [file pone.0049349.s003.tif]

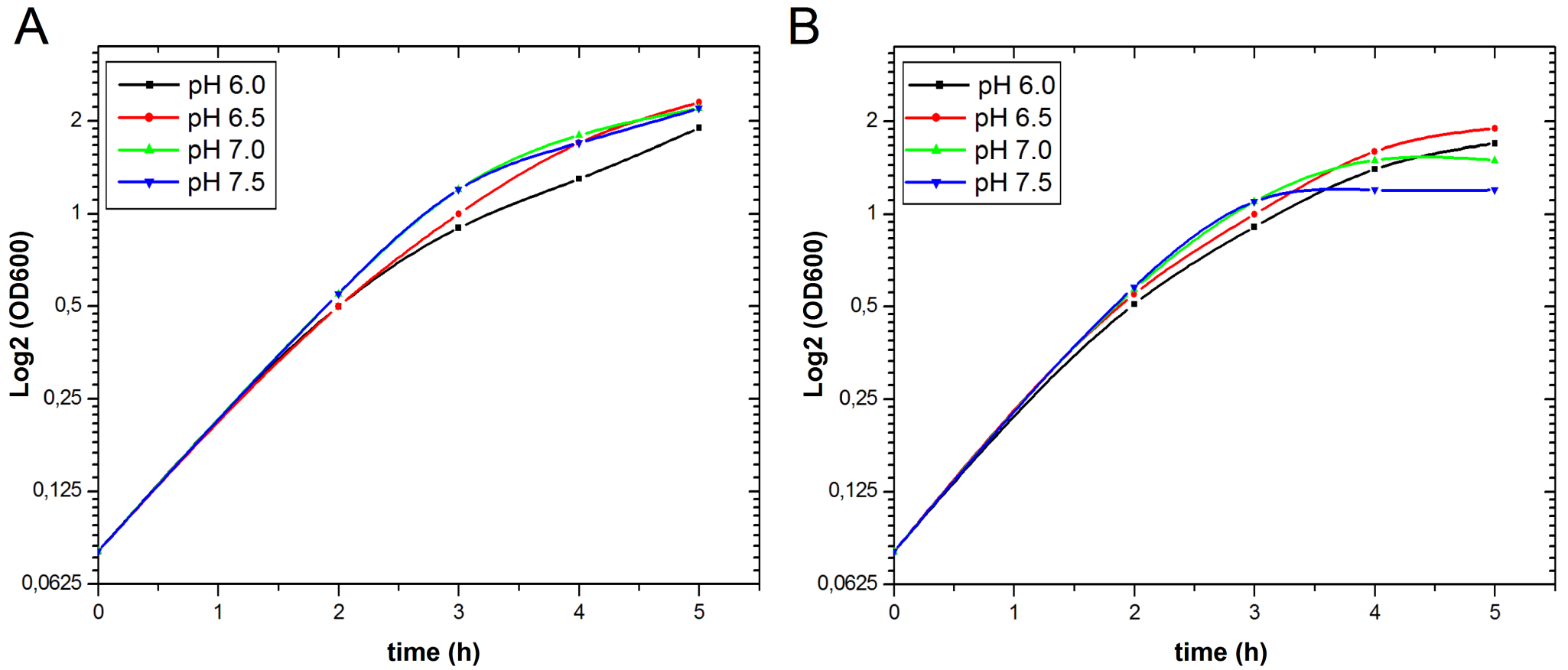

Supplement: Figure S4 — Y. pseudotuberculosis growth kinetics were pH independent between pH 6.0 and pH 7.5. Bacterial growth of wild-type Y. pseudotuberculosis was analyzed by monitoring the optical density (OD600) under (A) calcium-supplemented and (B) calcium-depleted conditions. Bacteria were cultivated 2 h at 26°C, then 3 h at 37°C. Samples were taken every hour to monitor the growth based on the measured OD600. No significant differences in growth kinetics were observed within the monitored pH-interval of 6.0 to 7.5. (TIF) [file pone.0049349.s004.tif]

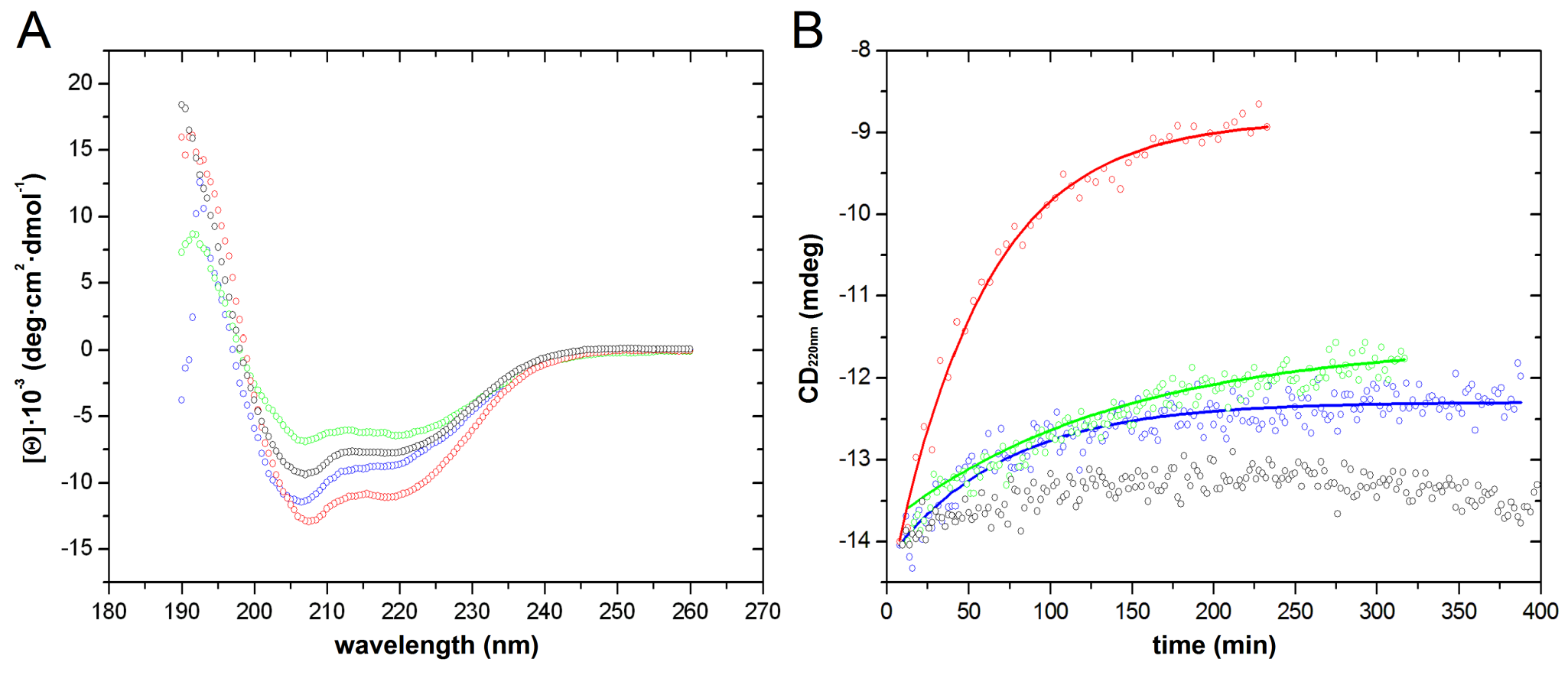

Supplement: Figure S5 — Secondary structure analysis and dissociation kinetics of YscUC suppressor mutants monitored with CD spectroscopy. (A) Far-UV CD spectra of 10 µM wild-type YscUC (black) and the suppressor mutants A268F (blue), Y287G (green), V292T (red). The similarity in the shape of the CD signals indicates that all YscUC variants have similar secondary structure. (B) Dissociation kinetics of YscUC (black) and suppressor mutants A268F (blue), Y287G (green), and V292T (red) were monitored with CD spectroscopy at 220 nm and 37°C. The solid lines represent the best fit of a single exponential decay function to determine τ diss. Dissociation kinetics are summarized in Table 3. Note that the data points at time = zero have been normalized for clarity. (TIF) [file pone.0049349.s005.tif]

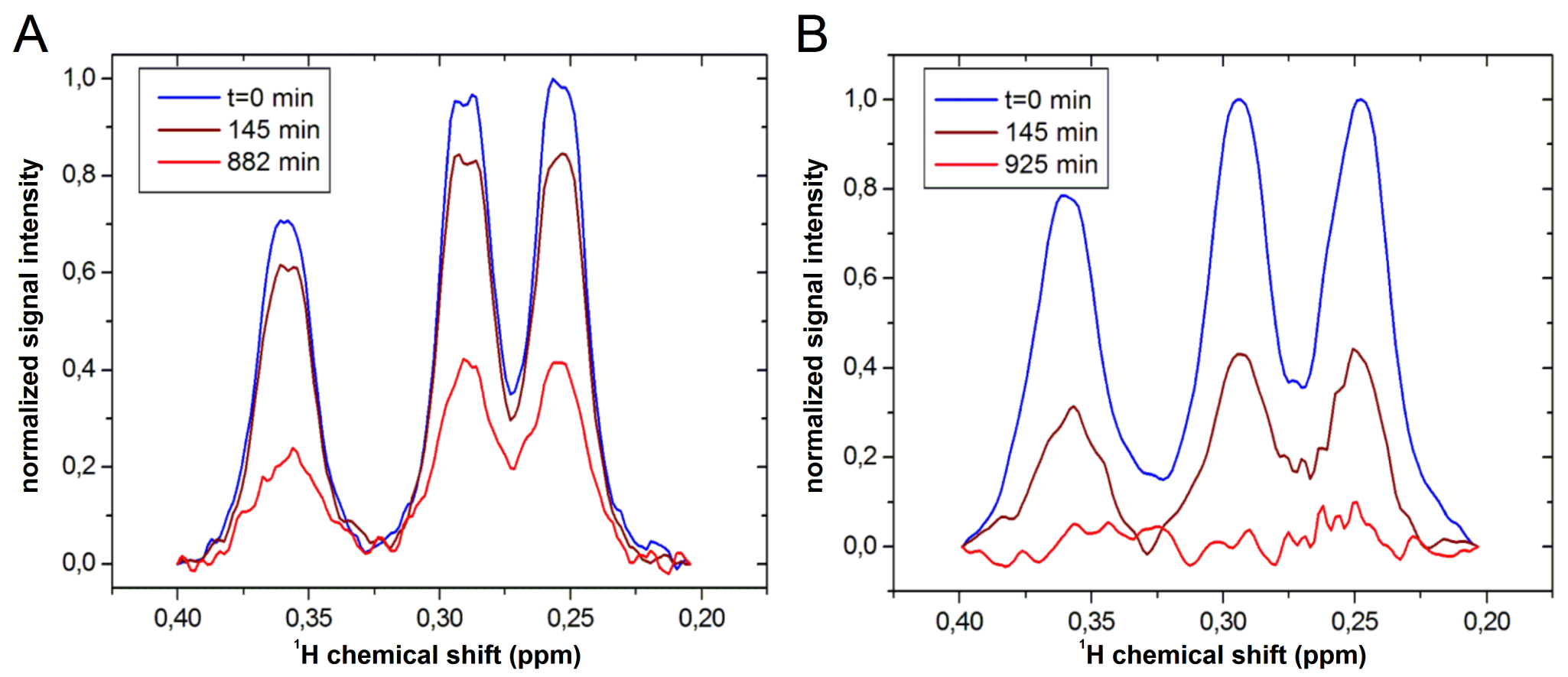

Supplement: Figure S6 — Primary NMR data used to quantify dissociation kinetics of wild-type YscUC and V292T at 37°C. Shown are expansions of methyl resonances from one-dimensional 1H spectra at various time points for (A) wild-type YscUC and (B) the V292T mutant (see Figure 7B and 7C). (TIF) [file pone.0049349.s006.tif]

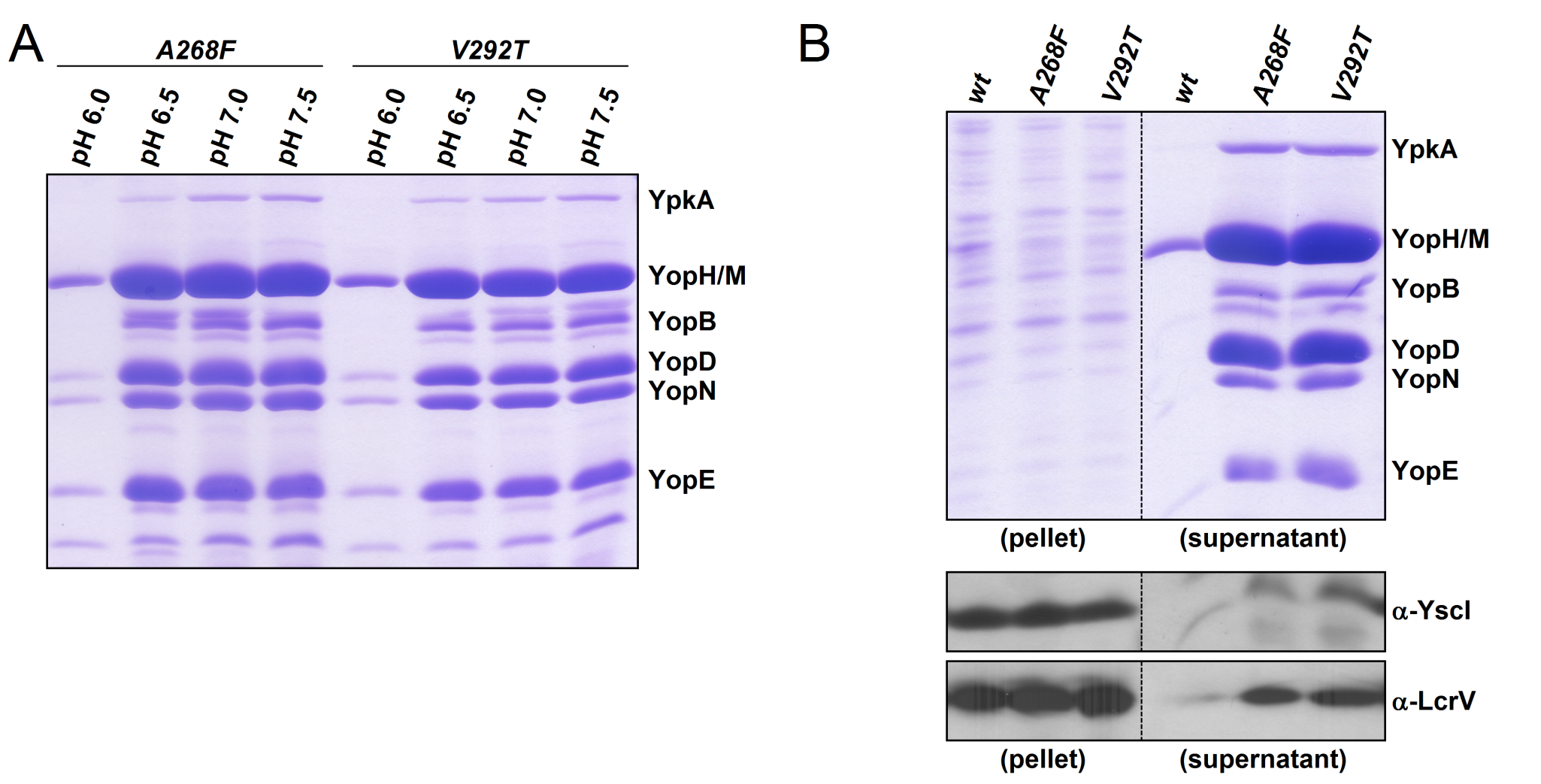

Supplement: Figure S7 — Secretion analysis of ysc U suppressor mutants A268F and V292T . (A) Secretion analysis of A268F and V292T after cultivation in Hepes-buffered LB at different pH values. Yop secretion was induced by calcium depletion and a temperature shift from 26°C to 37°C. A268F and V292T showed elevated Yop secretion at pH≥6.5 and strong inhibition at pH 6.0 (B) Secretion analysis of A268F and V292T at pH 7.5 and 30°C compared to wild-type. The T3SS was induced by calcium depletion and a temperature shift to 30°C. A268F and V292T showed a strongly elevation of Yop secretion. Coomassie stained gels demonstrate secreted Yops. “pellet” indicates intracellular proteins; “supernatant” denotes secreted proteins. The secretion of YscI and LcrV was visualized on immunoblots with anti-YscI and anti-LcrV antibodies. (TIF) [file pone.0049349.s007.tif]

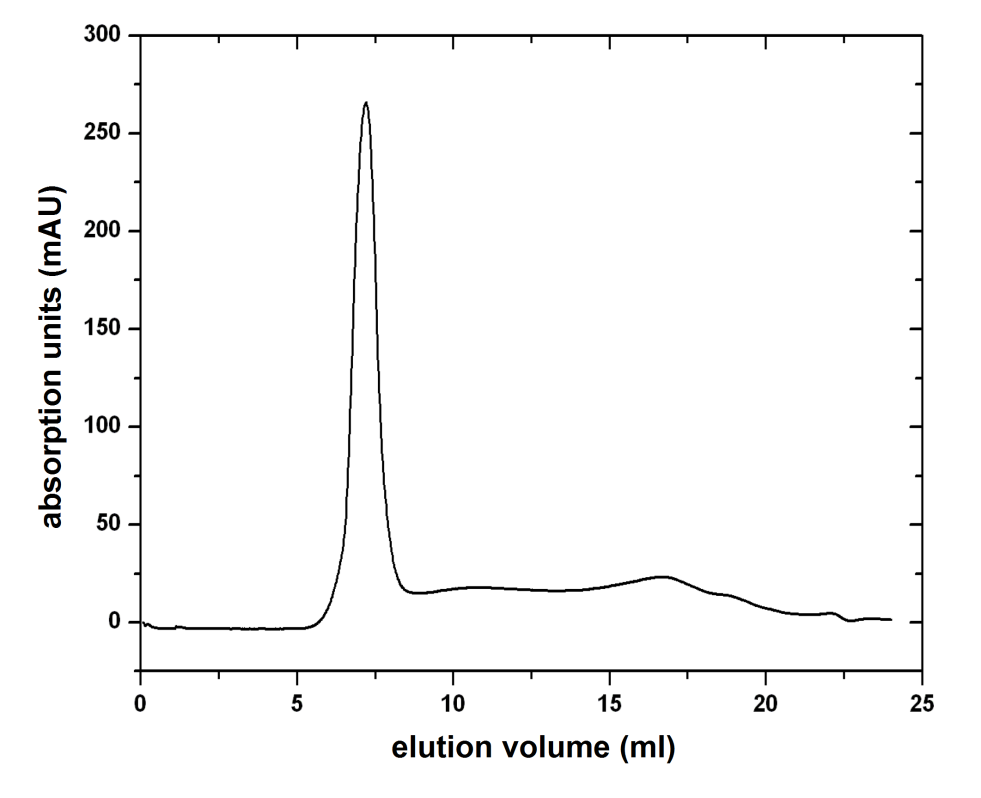

Supplement: Figure S8 — Size exclusion chromatography-based estimation of the YscUCC aggregate size. Chromatogram of analytical size exclusion chromatography of purified YscUCC. YscUCC eluted in the void volume of the SEC column. (TIF) [file pone.0049349.s008.tif]

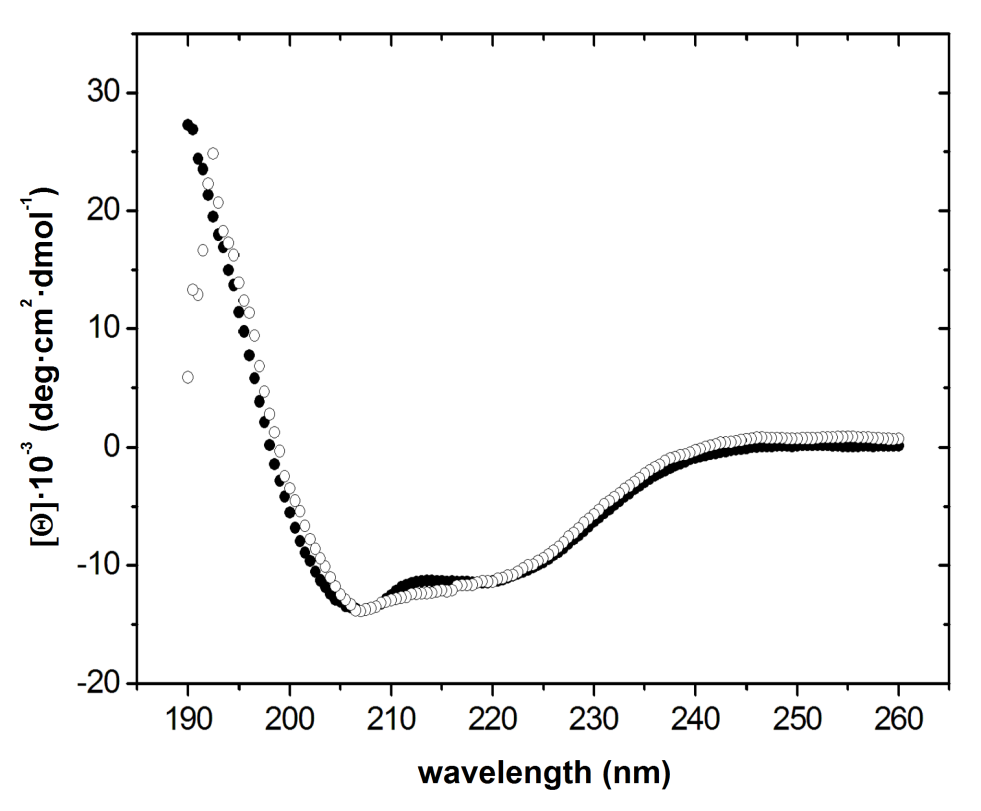

Supplement: Figure S9 — CD-based analysis of YscUCC produced by thermal stimulation or by recombinant protein production. Comparison of YscUC CD spectra at 20°C after one completed thermal cycle to 95°C. Filled circles show the YscUCC produced by thermal stimulation and open circles show the purified YscUCC fragment, produced in vitro by recombinant protein production. The similarity of the spectra shows that the residual CD signal of YscUC after one thermal cycle is dominated by the YscUCC fragment. (TIF) [file pone.0049349.s009.tif]
