# Supplementary material for: Autoproteolysis and Intramolecular Dissociation of Yersinia YscU Precedes Secretion of Its C-Terminal Polypeptide YscUCC
Source: PLoS One. 2012 Nov 21;7(11):e49349. doi: 10.1371/journal.pone.0049349 (PMC3504009; doi:10.1371/journal.pone.0049349)
Supplement: Table S1 — Bacterial strains and plasmids used in this study. (RTF) [file pone.0049349.s010.rtf]

Table S1: Bacterial strains and plasmids used in this study
Strains, plasmids, or constructs	Descriptiona	Reference	
E. coli strains			
   BL21 (DE3) pLysS	IPTG-inducible T7 RNA polymerase	Studier, Moffat	
   Top 10	Commercial one-shot competent cells	Invitrogen	
			
Y. pseudotuberculosis strains			
   YPIII(pIB102)	wild-type, parental strain, Kmr		
   YPIII(YscU)/pNQ	yscU, insertional mutant, Kmr Cmr	Björnfot et al., 2009	
   YPIII(A268F)/pNQ	yscU(A268F), insertional mutant, Kmr Cmr	Björnfot  et al., 2009	
   YPIII(V292T)/pNQ	yscU(V292T), insertional mutant, Kmr Cmr	Björnfot  et al., 2009	
   YPIII(pIB102)	ÄyscC deletion mutant, Kmr	Björnfot, unpublished	
   YPIII(pIB082)	ÄyopN deletion mutant, Kmr	Forsberg et al., 1991	
			
Plasmids			
   pGEX-6P3	Commercial vector with N-term. GST-fusion	GE Healthcare	
   pBADmycHis B	Commercial vector for L-ara induced expression	Invitrogen	
			
Constructs			
   GST-YscUC	YscUC in pGEX-6p-3	This study	
   GST-YscUCC	YscUCC in pGEX-6p-3	This study	
   YscUCC	YscUCC in pBADmycHisB	This study	
   GST-YscUC(A268F)	ÄyscP suppressor mutant of YscUC in pGEX-6p-3	This study	
   GST-YscUC(Y287G)	ÄyscP suppressor mutant of YscUC in pGEX-6p-3	This study	
   GST-YscUC(V292T)	ÄyscP suppressor mutant of YscUC in pGEX-6p-3	This study	
   GST-YscUC(H324A)	histidine mutant for pH-dependency in pGEX-6p-3  	This study	
   GST-YscUC-His6	GST-YscUC with C-term. His6-tag in pGEX-6p-3	This study	
   GST-A268F-His6	GST-YscUC(A268F) with C-term. His6-tag in 
pGEX-6p-3	This study	
a Kmr, kanamycin resistance; Cmr, chloramphenicol resistance; Cbr, carbenicillin resistance 	
