# Supplementary material for: Autoproteolysis and Intramolecular Dissociation of Yersinia YscU Precedes Secretion of Its C-Terminal Polypeptide YscUCC
Source: PLoS One. 2012 Nov 21;7(11):e49349. doi: 10.1371/journal.pone.0049349 (PMC3504009; doi:10.1371/journal.pone.0049349)
Supplement: Table S2 — Primers used in this study. (RTF) [file pone.0049349.s011.rtf]

Table S2: Primers used in this study
Primer name	Primer sequence (5'- 3')	Restriction sites	
Primers for sub-cloning		
fw_yscUC_pGEX	atggatccattaaggaacttaaaatgagcaag	BamHI	
rv_yscUC_pGEX	atgcggccgcttataacatttcggaatgttgtttctc	NotI	
fw_yscUCC_pGEX	atggatccccgacccatattgctattggt	BamHI	
fw_yscUCC_pBAD	atccatggatccgacccatattgctattgg	NcoI	
rv_yscUCC_pBAD	attctagattataacatttcggaatgttgtttctcg	XbaI	
fw_yscUCHis_pGEX	atggatccattaaggaacttaaaatgagcaaggatgagatcaaacgcg	BamHI	
rv_yscUCHis_pGEX	atgcggccgcttagtggtggtggtggtggtgctcgagtaacatttcggaatgttgtttctcg	NotI	
			
Primers for site-directed mutagenesis		
fw_yscUC_A268F	gtggtagctaatccgacccatatttttattggtattctttacaagcga	-	
rv_yscUC_A268F	tcgcttgtaaagaataccaataaaaatatgggtcggattagctaccac	-	
fw_yscUC_Y287G	caccactaccgttggtaacattcaaaggtaccgatgcccaag	-	
rv_yscUC_Y287G	cttgggcatcggtacctttgaatgttaccaacggtagtggtg	-	
fw_yscUC_V292T	cattcaaatataccgatgcccaaactcagactgtgcgcaaaatagc	-	
rv_yscUC_V292T	gctattttgcgcacagtctgagtttgggcatcggtatatttgaatg	-	
fw_yscUC_H324A	gcgctcgtcgatgcctatattccggctgagc	-	
rv_yscUC_H324A	gctcagccggaatataggcatcgacgagcgc	-	
