# Supplementary material for: Autoproteolysis and Intramolecular Dissociation of Yersinia YscU Precedes Secretion of Its C-Terminal Polypeptide YscUCC
Source: PLoS One. 2012 Nov 21;7(11):e49349. doi: 10.1371/journal.pone.0049349 (PMC3504009; doi:10.1371/journal.pone.0049349)
Supplement: Materials and Methods S1 — Procedure for cloning yscU CC into pBADmyc His B. (RTF) [file pone.0049349.s012.rtf]

Procedure for cloning yscUCC into pBADmyc His B
DNA was amplified by PCR from the Y. pseudotuberculosis strain, YPIII(pIB102). PCR amplifications were run with primer pairs, fw_yscUCC_pBAD/ rv_yscUCC_pBAD (see supporting material, “Table S2”). The PCR products were digested and ligated into the pBAD mycHis B vector, then transformed into Top10 cells for plasmid amplification. Subsequently, the purified plasmids were transformed into Y. pseudotuberculosis or E. coli BL21(DE3) pLysS strains for further analysis.
