# Supplementary material for: Autoproteolysis and Intramolecular Dissociation of Yersinia YscU Precedes Secretion of Its C-Terminal Polypeptide YscUCC
Source: PLoS One. 2012 Nov 21;7(11):e49349. doi: 10.1371/journal.pone.0049349 (PMC3504009; doi:10.1371/journal.pone.0049349)
Supplement: Materials and Methods S2 — Cloning procedure for GST fusion proteins. (RTF) [file pone.0049349.s013.rtf]

Cloning procedure for GST fusion proteins
DNA was amplified by PCR from the Y. pseudotuberculosis strain, YPIII(pIB102). The primer pairs used for PCR amplification are described in the supporting material (“Table S2”). PCR products were purified, digested, and ligated into the pGEX-6p-3 vector. To produce the suppressor mutants of yscUC, the pGEX-6p-3 vector carrying the yscUC DNA sequence served as a template. Point mutations were introduced with the QuikChange Site-Directed Mutagenesis Kit. All plasmids were amplified by transforming into Top10 cells. Subsequently, purified plasmids were transformed into E. coli BL21(DE3) pLysS for protein production. This procedure was used to obtain the following proteins: the entire YscU cytoplasmic tail (YscUC), the cleaved C-terminal YscU cytosolic domain (YscUCC), the His-tag modified entire YscU cytoplasmic tail (GST-YscUC-His), the mutant YscU(A268F), the mutant YscU(Y287G), the mutant YscU(V292T) and the mutant YscU(H324A).
