# Supplementary material for: Autoproteolysis and Intramolecular Dissociation of Yersinia YscU Precedes Secretion of Its C-Terminal Polypeptide YscUCC
Source: PLoS One. 2012 Nov 21;7(11):e49349. doi: 10.1371/journal.pone.0049349 (PMC3504009; doi:10.1371/journal.pone.0049349)
Supplement: Results S1 — YscUCC aggregated after dissociation from YscUCN. Analytical size exclusion chromatography was used to show that YscUCC aggregates after dissociation from the YscUCN polypeptide. (RTF) [file pone.0049349.s014.rtf]

YscUCC aggregated after dissociation from YscUCN
NMR spectroscopy is not suitable for very large proteins that tumble slowly in solution; therefore, the absence of a signal that corresponded to the YscUCC polypeptide after heat treatment suggested that this polypeptide had formed an aggregate too large for detection with NMR. Aggregation of YscUCC was verified by estimating the apparent molecular weight of the recombinant, purified YscUCC with analytical size exclusion chromatography (SEC) (Figure S8). We found that YscUCC migrated in the void volume of the SEC column, which indicated that YscUCC formed a large, soluble aggregate. Hence, YscUCC aggregation explained the absence of signals in the NMR experiment. It should be noted that incubation of YscUCC for prolonged time periods at 60 °C resulted in complete precipitation of the polypeptide. The aggregation/precipitation behavior of YscUCC was most likely an in vitro property triggered by the high concentrations used in the biophysical measurements. Accordingly, we do not suggest that YscUCC aggregation is relevant in vivo.
