# Supplementary material for: Autoproteolysis and Intramolecular Dissociation of Yersinia YscU Precedes Secretion of Its C-Terminal Polypeptide YscUCC
Source: PLoS One. 2012 Nov 21;7(11):e49349. doi: 10.1371/journal.pone.0049349 (PMC3504009; doi:10.1371/journal.pone.0049349)
Supplement: Results S2 — Persistent secondary structure in aggregated YscUCC from CD spectroscopy. CD spectroscopy on thermally and recombinantly produced YscUCC aggregates was used to show that YscUCC contains elements of secondary structure in the aggregated state. (RTF) [file pone.0049349.s015.rtf]

Persistent secondary structure in aggregated YscUCC from CD spectroscopy
A strong CD signal was observed after YscUC was subjected to a thermal cycle (Figure 2B). This result indicated that there were persistent secondary structure elements in the YscUCC aggregate, because, under these conditions, NMR spectroscopy showed that YscUCN was unfolded. To show directly that the residual CD signal after the thermal cycle was dependent on the YscUCC fragment structure, we compared the CD spectrum (at 20 °C) of YscUC after a thermal cycle to the spectrum of an YscUCC peptide produced with recombinant protein production (Figure S9). The spectra were strikingly similar. Thus, we concluded that the residual CD signal depended on elements of secondary structure in the YscUCC aggregate.
